# Comprehensive bioinformatic analysis reveals a cancer-associated fibroblast gene signature as a poor prognostic factor and potential therapeutic target in gastric cancer

**DOI:** 10.1186/s12885-022-09736-5

**Published:** 2022-06-23

**Authors:** Cemre Ucaryilmaz Metin, Gulnihal Ozcan

**Affiliations:** 1 Graduate School of Health Sciences, Koc University, 34450 Istanbul, Turkey; 2 Department of Medical Pharmacology, Koc University School of Medicine, 34450 Istanbul, Turkey

**Keywords:** Gastric cancer, Cancer-associated fibroblasts, Extracellular matrix, Tumor microenvironment, Bioinformatics, Prognostic biomarkers, Therapeutic targets

## Abstract

**Background:**

Gastric cancer is one of the deadliest cancers, currently available therapies have limited success. Cancer-associated fibroblasts (CAFs) are pivotal cells in the stroma of gastric tumors posing a great risk for progression and chemoresistance. The poor prognostic signature for CAFs is not clear in gastric cancer, and drugs that target CAFs are lacking in the clinic. In this study, we aim to identify a poor prognostic gene signature for CAFs, targeting which may increase the therapeutic success in gastric cancer.

**Methods:**

We analyzed four GEO datasets with a network-based approach and validated key CAF markers in The Cancer Genome Atlas (TCGA) and The Asian Cancer Research Group (ACRG) cohorts. We implemented stepwise multivariate Cox regression guided by a pan-cancer analysis in TCGA to identify a poor prognostic gene signature for CAF infiltration in gastric cancer. Lastly, we conducted a database search for drugs targeting the signature genes.

**Results:**

Our study revealed the *COL1A1, COL1A2, COL3A1, COL5A1, FN1*, and *SPARC* as the key CAF markers in gastric cancer. Analysis of the TCGA and ACRG cohorts validated their upregulation and poor prognostic significance. The stepwise multivariate Cox regression elucidated *COL1A1* and *COL5A1*, together with *ITGA4, Emilin1*, and *TSPAN9* as poor prognostic signature genes for CAF infiltration. The search on drug databases revealed collagenase *clostridium histolyticum*, ocriplasmin, halofuginone, natalizumab, firategrast, and BIO-1211 as the potential drugs for further investigation.

**Conclusions:**

Our study demonstrated the central role of extracellular matrix components secreted and remodeled by CAFs in gastric cancer. The gene signature we identified in this study carries high potential as a predictive tool for poor prognosis in gastric cancer patients. Elucidating the mechanisms by which the signature genes contribute to poor patient outcomes can lead to the discovery of more potent molecular-targeted agents and increase the therapeutic success in gastric cancer.

**Supplementary Information:**

The online version contains supplementary material available at 10.1186/s12885-022-09736-5.

## Background

Gastric cancer is the fifth most common cancer worldwide and the fourth leading cause of cancer-related deaths, the GLOBOCAN 2020 statistics report. More than one million people were diagnosed with gastric cancer, and more than 750,000 deaths occurred due to gastric cancer in 2020 [1]. Stomach adenocarcinomas (STAD) constitute almost 95% of all gastric cancer cases. The mainstay of treatment in localized stomach adenocarcinoma is gastrectomy with total lymphadenectomy and chemotherapy [2]. However, the tumors are commonly metastatic at the time of diagnosis. At this stage, complete resection is impossible, and currently available chemotherapeutics fail due to chemoresistance [2, 3].

Molecular-targeted agents against tumor-specific biomarkers and immunotherapy increased the treatment efficacy in certain cancers such as breast cancer, lung cancer, and melanoma [4]. However, similar success is not achieved in gastric cancer yet. Currently, molecular-targeted agents, nivolumab (anti-PD-1), pembrolizumab (anti-PD-1), ramucirumab (anti-VEGFR2), and trastuzumab (anti-HER2), are approved in gastric cancer treatment. Unfortunately, they have limited efficacy on the overall survival of a limited group of advanced-stage patients with target positivity [5].

The tumor microenvironment is a great challenge for the treatment of cancer. Dynamic interactions with the extracellular matrix (ECM) and cellular components in the tumor microenvironment potentiate the aggressiveness of cancer cells and limit their response to anti-cancer agents [6]. Cancer-associated fibroblasts (CAFs) are critical components in the cellular compartment of the tumor microenvironment that assemble and remodel the ECM. They originate from activated fibroblasts and the endothelial or epithelial cells undergoing epithelial-mesenchymal transition (EMT). They secrete various ECM proteins and soluble mediators that potentiate pro-tumorigenic signaling pathways via activation of transmembrane receptors – mainly integrins. Moreover, CAFs remodel the ECM to form a protective barrier against immune surveillance and the diffusion of anti-cancer agents [7]. CAF infiltration is associated with a dismal prognosis in gastric cancer [8, 9]. CAFs were identified as the greatest risk factor in tumor microenvironment phenotype with the poorest overall survival in gastric cancer patients [10]. Therefore, addressing CAFs is essential for the treatment of gastric cancer. However, the poor prognostic markers for CAF infiltration in gastric cancer are not clear and drugs that target CAF-mediated processes are lacking in the clinic.

**Fig. 1:**
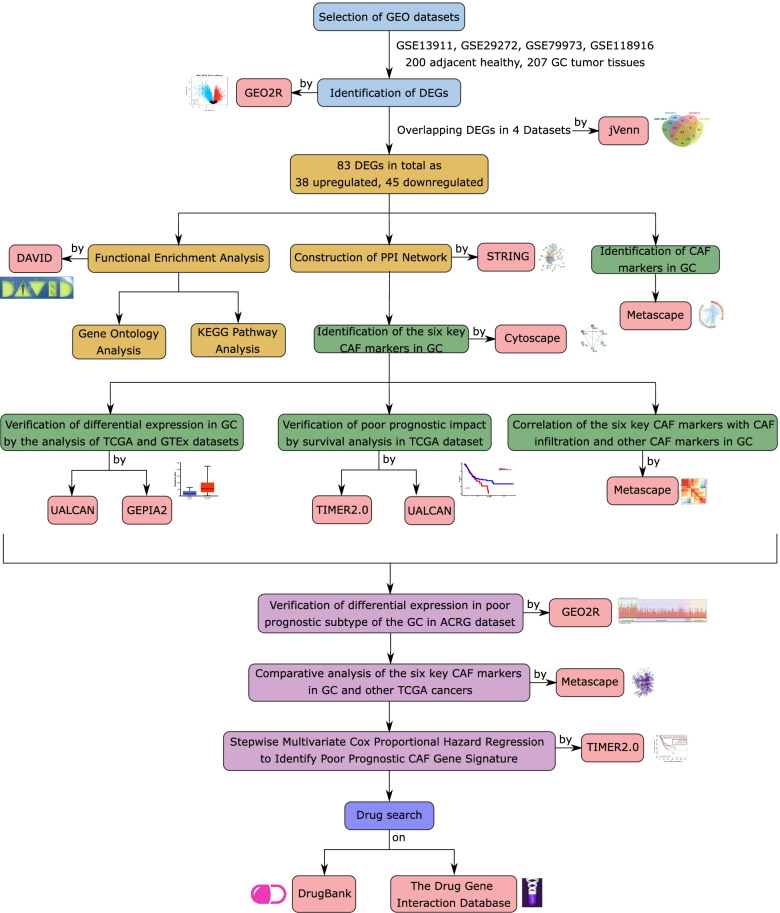
The workflow in the study. GSE13911, GSE29272, GSE79973, and GSE118916 denote four expression profiling datasets from the GEO database. ACGR: Asian Cancer Research Group, CAF: cancer-associated fibroblast, DAVID: The Database for Annotation, Visualization, and Integrated Discovery, DEG: differentially expressed genes, GC: Gastric cancer, GEPIA2: Gene Expression Profiling Interactive Analysis 2, GTEx: Genotype-Tissue Expression Project, KEGG: Kyoto Encyclopedia of Genes and Genomes, PPI: protein-protein interaction, STRING: The Search Tool for the Retrieval of Interacting Genes/Proteins, TCGA: The Cancer Genome Atlas, TIMER2.0: Tumor Immune Estimation Resource 2.0, UALCAN: University of Alabama Cancer Database

In this study, we aim to identify the poor prognostic gene signature for CAF infiltration targeting which may increase the therapeutic efficacy in a large group of gastric cancer patients. We comprehensively analyzed four gastric cancer GEO gene expression datasets using a network-based approach and identified key markers for CAF infiltration. After validation of the markers in The Cancer Genome Atlas (TCGA) and Asian Cancer Research Group (ACRG) cohorts, we elucidated a poor prognostic gene signature for CAF infiltration in gastric cancer using stepwise multivariate Cox regression. Lastly, we compiled the list of currently available drugs that may have a therapeutic potential in gastric cancer by targeting the signature genes we identified (Fig. 1 summarizes the steps followed in the study).

## Methods

### Data collection and identification of differentially expressed genes in gastric cancer

We analyzed four GEO expression profiling datasets (GSE13911, GSE29272, GSE79973, GSE118916) [11–14], which used Affymetrix Human Genome or Gene Expression Arrays for profiling (https://www.ncbi.nlm.nih.gov/geo/). All four datasets bear a comparable number of samples from gastric cancer tissues and non-cancerous gastric tissues. Additional file 1: Table S1 lists the number of tissue samples and the profiling platforms in each dataset. In total, we analyzed 200 non-tumor gastric and 207 gastric tumor samples. To identify differentially expressed genes (DEGs) in gastric tumors compared to non-cancerous stomach samples, we used the GEO2R web tool (https://www.ncbi.nlm.nih.gov/geo/geo2r/). We applied log transformation and Benjamini & Hochberg’s (False discovery rate) method to adjust the *p*-values (*p*-value significance cut-off = 0.01). The genes were filtered based on their log2-fold change (logFC) values. We accepted the genes with the log FC value >1 as the upregulated genes and with the log FC value < −1 as the downregulated genes. After identifying the DEGs in each dataset, we performed Venn Analysis to find DEGs common to all four datasets using the jvenn (an interactive Venn diagram viewer) (http://jvenn.toulouse.inra.fr/app/index.html).

### Functional annotation and enrichment analysis

To perform functional enrichment and annotation clustering analysis of the DEGs, we used The Database for Annotation, Visualization, and Integrated Discovery (DAVID) (Version 6.8) [15] (https://david.ncifcrf.gov/). To understand the cellular compartments (GO-CC), molecular functions (GO-MF), and biological processes (GO-BP) at which the DEGs enriched, we performed gene ontology (GO) analysis. To understand the pathways at which the DEGs operate, we performed the Kyoto Encyclopedia of Genes and Genomes (KEGG) pathway analysis [16]. The cut-off value for significance was chosen as *p* < 0.05. We analyzed gene lists for upregulated genes, downregulated genes, and all DEGs separately.

To compare the gene identities and gene ontologies enriched in different lists, we used Metascape [17] (https://metascape.org). To dissect the similarities and dissimilarities between gene lists, we analyzed the Circos plots, clustering dendrograms, network layouts for enriched gene ontologies, the proportion of the genes from different lists that fall into the same gene ontologies, and enrichment *p*-values.

### Protein-protein interaction network analysis

To identify the central CAF markers and assess their potential connections and interactions with the protein products of other DEGs in gastric cancer, we constructed the protein-protein interaction (PPI) network of the DEGs using The Search Tool for the Retrieval of Interacting Genes/Proteins (STRING Version 11.0) [18] (https://string-db.org/). We utilized the minimum required interaction score as high confidence (0.7). We analyzed the resulting PPI network in Cytoscape (Version 3.8.2) to infer the topological parameters of each node [19] (https://cytoscape.org/). To investigate the network modules, we used Molecular Complex Detection (MCODE) plugin at Cytoscape. To construct a local network for key CAF markers and their first neighbors, we used the Cytohubba plugin at Cytoscape [20, 21]. To investigate the interactors of ITGA4 we searched inBio Discover™ by Intomics A/S (https://inbio-discover.com/) (Intomics A/S has not endorsed the results of the published article) [22].

### Gene expression profiling

To confirm the differential expression of the CAF markers in gastric cancer, we comparatively analyzed the gene expression profiles of 34 non-cancerous gastric tissues and 415 stomach adenocarcinoma samples in the TCGA dataset using the UALCAN (University of Alabama Cancer Database) (http://ualcan.path.uab.edu/) [23]. We also investigated the differential expression of the CAF markers by tumor grade and stage (the unpaired t-test was used for statistical analysis). To validate the results from the UALCAN, we analyzed The Genotype-Tissue Expression Project (GTEx) data on GEPIA2 [24] (http://gepia2.cancer-pku.cn).

To investigate the differential expression of CAF markers in diffuse vs. intestinal subtypes and mesenchymal vs. epithelial phenotypes of gastric adenocarcinoma, we analyzed the ACRG cohort on GEO2R (GSE66229) [25]. We used the same set of parameters to identify DEGs in GSE66229 as for the datasets GSE13911, GSE29272, GSE79973, and GSE118916. Then we analyzed the expression profile graphs of all patients for the six CAF markers. To investigate the differential expression of CAF markers in other cancers we analyzed the TCGA data on Tumor Immune Estimation Resource 2.0 (TIMER2.0) [26] (https://timer.cistrome.org). Then, we extracted the pan-cancer expression profile graphs for CAF markers.

### Survival analysis

To understand the impact of the CAF markers on the survival of gastric cancer patients, we performed the Kaplan-Meier (KM) survival analysis of TCGA stomach adenocarcinoma samples on TIMER 2.0. The stomach adenocarcinoma samples split into high or low expression groups based on the median expression level for each gene. Additionally, KM-Survival Curve for COL1A2 was extracted from the UALCAN to assess its prognostic role in gastric cancer. We generated the heatmaps that show the z-scores for each gene in distinct cancers using the “gene outcome” module in TIMER 2.0. We constituted the KM-survival curves for CAF infiltration that integrate gene expression data with the “immune association” tool in TIMER 2.0, which utilizes the log-rank test. Then we extracted the hazard ratios (HR), z-scores, and *p*-values from the multivariate Cox proportional hazard regression models built on TIMER 2.0.

### Gene correlation analysis

We investigated the correlation between the individual gene expression and CAF infiltration in different cancers by the “immune association” tool in TIMER 2.0. To examine the correlation between distinct genes, we used the “Gene Correlation” module in TIMER 2.0, which gives purity adjusted correlation coefficients calculated by partial spearman rank correlation. We extracted the correlation coefficients and the *p*-values from TIMER2.0. to generate a gene correlation heatmap on the Bioinfo Intelligent Cloud (BIC) imageGP tool (http://www.ehbio.com/Cloud_Platform/front/#/). For hierarchical clustering of the heatmap, we selected the Spearman method on the imageGP.

### Potential drug search

To identify potential drugs that interfere with the target genes or proteins, we searched the DrugBank (https://www.drugbank.com/) and the Drug-Gene Interaction database (DGIdb v4.2.0) [27] (https://www.dgidb.org/).

### Data visualization

To draw the bubble plots for enriched ontology terms, we used the BIC imageGP tool. To plot the gene expression profiles and hazards ratios for CAF infiltration and gene expression in different cancers, we used GraphPad Prism9.

## Results

### Identification of cancer-associated fibroblast markers in gastric cancer

**Fig. 2:**
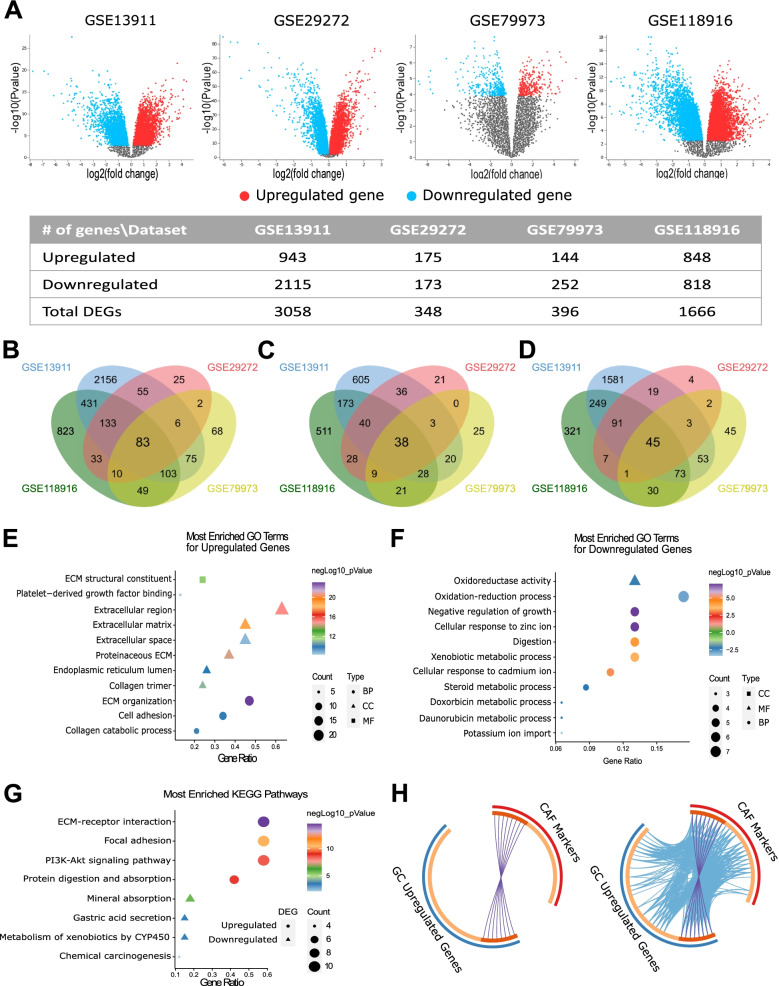
Identification and functional enrichment analysis of differentially expressed genes in gastric cancer. **A** Volcano plots of differentially expressed genes (DEGs) in four GEO datasets with the number of upregulated, downregulated genes and, the total number of DEGs detected in each dataset. Overlapping **B** DEGs, **C** upregulated genes, and **D** downregulated genes in four GEO datasets. Bubble plots of the most enriched GO terms for **E** upregulated genes, **F** downregulated genes, and **G** KEGG pathways for all DEGs (BP: GO-biological process, MF: GO-molecular function, and CC: GO-cellular compartment). **H** The circos plots show how genes from the CAF markers (27 genes, red outer arc) and upregulated genes in GC (38 genes, blue outer arc) lists overlap. On the inside, each arc represents a gene list, where each gene has a spot on the arc. The dark orange color represents the genes that appear in both lists and the light orange color represents genes that are unique to a single gene list. Purple lines (upper circos plot) link the same genes that are shared by the two lists. Blue lines (lower circos plot) link the different genes which fall into the same ontology term. The circos plots were prepared on Metascape.org

To identify the CAF markers in gastric cancer we first investigated the DEGs in gastric cancer vs. non-cancerous gastric tissues. We analyzed GSE13911, GSE29272, GSE79973, and GSE118916 datasets which included microarray data for gastric tumors of various subtypes. To determine the most relevant markers, we applied strict criteria for the identification of the DEGs. We accepted genes with a log FC value of >1 or < −1 and an adjusted *p*-value <0.01, rather than accepting all genes with a log FC different than zero and *p*-value <0.05 as DEGs. Figure 2a shows the volcano plots and the number of the DEGs we detected in each dataset. The four datasets shared 83 DEGs: 38 upregulated- and 45 downregulated- genes (Fig. 2b-d; Additional file 1: Table S2).

To illuminate the biological functions and the pathways the DEGs enrich, we performed the functional annotation and enrichment analysis of 83 DEGs. The functional annotation clustering with the highest classification stringency in DAVID revealed 3 clusters (Table 1). The cluster with the highest enrichment score included the ECM-receptor interaction, focal adhesion, and PI3K-Akt signaling pathway.

**Table 1:** Functional annotation clustering of 83 differentially expressed genes in gastric cancer. Analysis was performed with the highest classification stringency in DAVID (ECM: Extracellular Matrix, GOTERM: Gene Ontology Term, GOTERM BP: GO-biological process, GOTERM MF: GO-molecular function, and GOTERM CC: GO-cellular compartment, KEGG Pathway: Pathways listed in Kyoto Encyclopedia of Genes and Genomes)

**Annotation Cluster 1**	**Enrichment Score: 7.26**	**Count**	**Genes**	***P*-Value**	**Benjamini**
KEGG Pathway	ECM-receptor interaction	12	*COL1A1, COL1A2, COL2A1, COL3A1, COL4A1, COL5A1, COL5A2, COL6A3, FN1, SPP1, THBS1, THBS2*	2.2E-11	1.6E-9
KEGG Pathway	Focal adhesion	12		2.2.E-7	5.2E-6
KEGG Pathway	PI3K-Akt signaling pathway	12		3.4E-5	4.0E-4
**Annotation Cluster 2**	**Enrichment Score: 6.68**	**Count**		***P*-Value**	**Benjamini**
GOTERM BP DIRECT	Negative regulation of growth	6	*MT1E, MT1F, MT1G,* *MT1H, MT1M, MT1X*	2.4E-8	3.1E-6
GOTERM BP DIRECT	Cellular response to zinc ion	6		2.4E-8	3.1E-6
KEGG Pathway	Mineral absorption	6		1.6E-5	2.3E-4
**Annotation Cluster 3**	**Enrichment Score: 1.25**	**Count**		***P*-Value**	**Benjamini**
GOTERM MF DIRECT	Oxygen binding	3	*CYP2C18, CYP2C9, CYP3A5*	2.0E-2	2.4E-1
GOTERM MF DIRECT	Oxidoreductase activity, acting on paired donors with incorporation or reduction of molecular oxygen	3		2.9E-2	2.9E-1
GOTERM MF DIRECT	Monooxygenase activity	3		2.9E-2	2.9E-1
GOTERM CC DIRECT	Organelle membrane	3		5.9E-2	3.2E-1
KEGG Pathway	Retinol metabolism	3		8.1E-2	4.8E-1
GOTERM MF DIRECT	Heme binding	3		1.3E-1	6.7E-1
GOTERM MF DIRECT	Iron ion binding	3		1.6E-1	7.8E-1

Functional enrichment analysis of the upregulated genes indicated a key role in ECM organization, ECM remodeling, ECM-receptor interaction, and activation of pro-tumorigenic signaling pathways (Fig. 2e, Additional file 1: Table S3). The most enriched molecular functions were ECM structural constituent, platelet-derived growth factor binding, and integrin-binding. The top KEGG pathways related to the upregulated genes were ECM-receptor interaction, focal adhesion, and PI3K-Akt signaling (Fig. 2g, Additional file 1: Table S3). The downregulated genes enriched in metabolic processes and ion homeostasis (Fig. 2f-g, Additional file 1: Table S4). These findings pointed out the upregulation of the ECM organization and remodeling in gastric cancer and suggested the involvement of CAFs.

To determine the CAF markers upregulated in gastric cancer we comparatively analyzed the list of upregulated DEGs in gastric cancer with an extended list of CAF markers, in terms of identity and gene ontology in Metascape. The CAF markers list included 27 genes: 18 commonly used CAF markers (*ACTA2, COL5A1, COL16A1, EMILIN1, FAP, FOXF1, LOXL1, LUM, MMP2, MMP11, PDGFRA, PDGFRB, PDPN, S100A4, SLC16A4, SPARC, VIM, ZEB1*) and 9 CAF-specific markers (*ASPN, COL1A1, COL1A2, COL3A1, COL11A1, FN1, MFAP5, OGN, TNC*). Eight out of 38 upregulated genes in gastric cancer (*ASPN, COL1A1, COL1A2, COL3A1, COL5A1, FAP, FN1*, and *SPARC*) overlapped with the CAF markers list (Fig. 2h, circos plot on the left). Besides that, 28 out of 38 upregulated genes in gastric cancer and 23 out of 27 CAF markers fell into the same ontology term that is statistically significantly enriched in both lists (Fig. 2h, circos plot on the right).

### Protein-protein interaction network analysis and identification of the key CAF markers

**Fig. 3:**
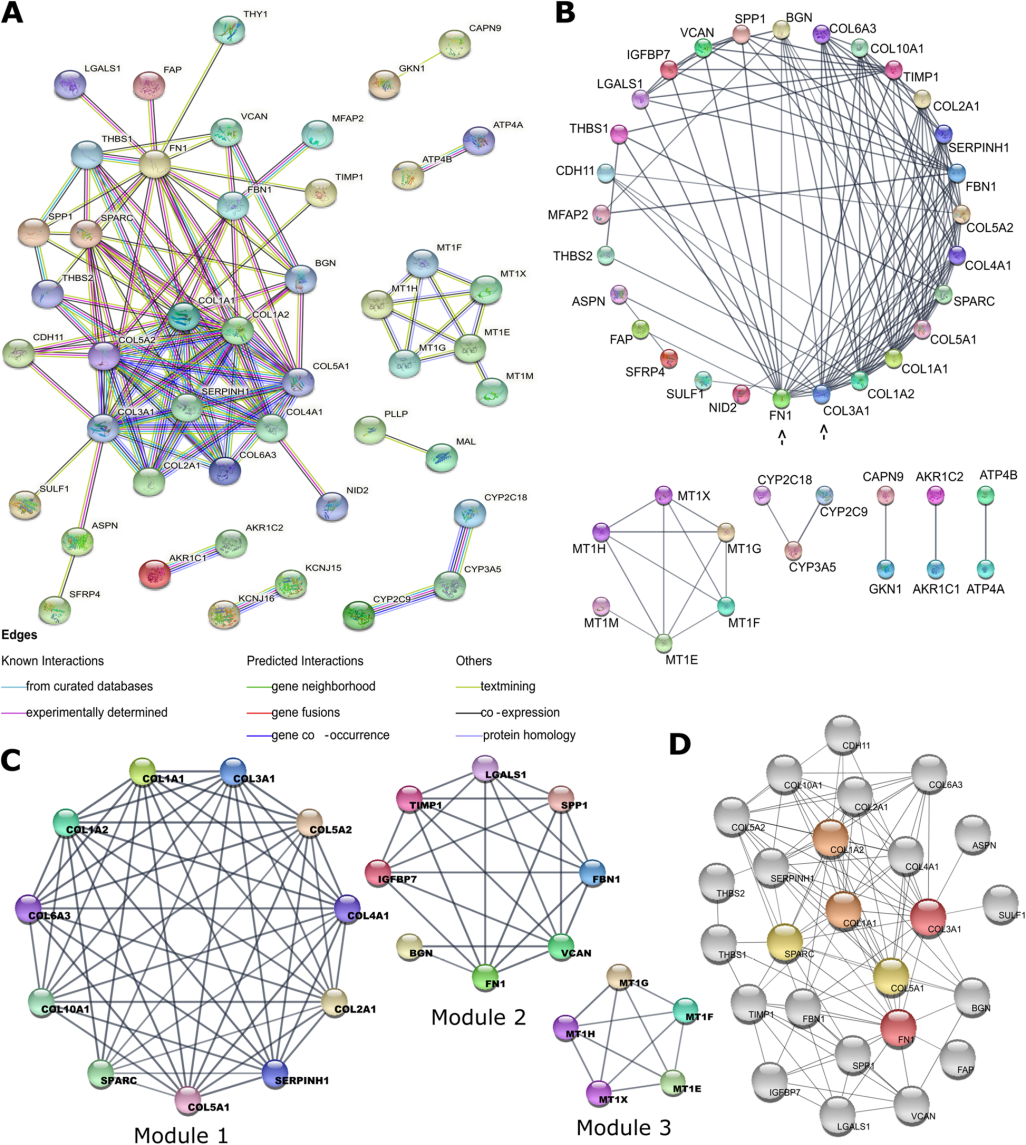
Protein-protein interaction (PPI) network of 83 differentially expresses genes in gastric cancer. **A** PPI network graph constructed in STRING. (Line colors indicate the type of interaction evidence; edges indicate functional and physical protein associations. Minimum required interaction score: high confidence (0.7). Disconnected nodes are hidden in the network). **B** Degree sorted circular network graph constructed in Cytoscape. Black arrows show the two genes with the highest degree in the network. The degrees of the nodes decrease counterclockwise. **C** Modules in the protein-protein interaction network of 83 differentially expressed genes in gastric cancer. Module analysis was performed using the MCODE tool in Cytoscape. The scores, number of nodes, and edges for each module are listed in the **D** Neighbors of the six CAF markers *COL1A1* (orange), *COL1A2* (orange), *COL3A1* (red)*, COL5A1* (yellow)*, FN1*(red), and *SPARC* (yellow). The network was constructed using the Cytohubba tool in Cytoscape (Color grade from red to yellow indicates the descending order of node degrees in the network)

To identify the key CAF markers and investigate their interactions with the protein products of DEGs in gastric cancer, we constructed a PPI network in STRING (Fig. 3a). The analysis of this network on Cytoscape 3.8.2. revealed a prominent hub composed almost totally of ECM components and CAF markers. The top six proteins with the highest degree in this hub were all CAF markers: COL3A1 (collagen type III alpha 1 chain), FN1 (fibronectin 1), COL1A2 (collagen type I alpha 2 chain), COL1A1 (collagen type I alpha 1 chain), COL5A1 (collagen type V alpha 1 chain), and SPARC (cysteine-rich acidic matrix-associated protein) respectively (Fig. 3b). Table 2 shows the topological parameters for these markers. The topological parameters for the whole PPI network are listed in Additional file 1: Table S5. The components of the hub were all upregulated genes in gastric tumors, except for COL2A1 (collagen type II alpha 1 chain) (Additional file 2: Fig. S1). These findings strengthened the central role of CAF markers in gastric cancer.

**Table 2:** Topological parameters for the six central CAF markers

Gene Symbol	Gene	Degree	Closeness of Centrality	Clustering Coefficient	Average Shortest Path Length	Neighborhood Connectivity
***COL3A1***	collagen type III alpha 1 chain	16	0.71	0.52	1.41	10.12
***FN1***	fibronectin 1	16	0.69	0.43	1.44	9.68
***COL1A2***	collagen type I alpha 2 chain	15	0.68	0.59	1.48	10.80
***COL1A1***	collagen type I alpha 1 chain	15	0.68	0.62	1.48	11.26
***COL5A1***	collagen type V alpha 1 chain	13	0.64	0.76	1.56	12.15
***SPARC***	secreted protein acidic and cysteine rich	13	0.64	0.58	1.56	11.38

### Analysis of the network modules CAF markers involved

To identify the modules that the six key CAF markers function, we used the MCODE tool in Cytoscape. MCODE identified three modules. The upregulated ECM protein hub was represented by modules 1 and 2 in Fig. 3c. Five out of 6 CAF markers, COL1A1, COL1A2, COL3A1, COL5A1, and SPARC, were components of module 1, while the FN1 was in module 2.

Then we performed functional enrichment analysis to identify enriched KEGG pathways at each module. “ECM-receptor interaction” and “focal adhesion” were the common enriched KEGG pathways in modules 1 and 2 (Additional file 1: Table S6). “PI3K-Akt signaling pathway” was the third enriched pathway in module 1. Hence the module analysis strengthened the connection of the six CAF markers: *COL1A1, COL1A2, COL3A1, COL5A1, FN1* and, *SPARC*, with the 3 KEGG pathways: ECM-receptor interaction, focal adhesion and, PI3K-Akt signaling pathway in gastric cancer.

COL1A1, COL1A2, COL3A1, COL5A1, and FN1 are abundant structural proteins at the ECM. COL1A1 and COL1A2 are produced mainly by fibroblasts and together constitute the type I collagen in the connective tissue. COL3A1 and COL5A1 are the alpha-1 chains of type III and V collagen, which are found in connective tissue together with type I collagen [28, 29]. FN1 is a glycoprotein involved in cell adhesion, wound healing, and metastasis. Besides their structural role, these proteins bind to the integrins on the cell membrane, and through focal adhesion kinases, they activate intracellular signaling pathways such as PI3K-Akt and MAPK pathways [30]. SPARC encodes the cysteine-rich acidic matrix-associated protein that is an essential protein for ECM remodeling. It binds to collagens and fibronectin; and regulates the interactions of cells with the ECM [31].

We analyzed the first neighbors of these CAF markers in our PPI network using the Cytohubba tool in Cytoscape. All these CAF markers highly interacted with other structural ECM components: BGN (biglycan), THBS1/2 (thrombospondin 1/2), and VCAN (versican), or ECM remodeling enzymes like SERPINH1 (serpin family H member 1), SULF1 (sulfatase 1), and TIMP1 (tissue inhibitor matrix metalloproteinase 1) (Fig. 3d).

### The correlation of the six CAF markers with CAF infiltration and other CAF markers in gastric cancer

**Fig. 4:**
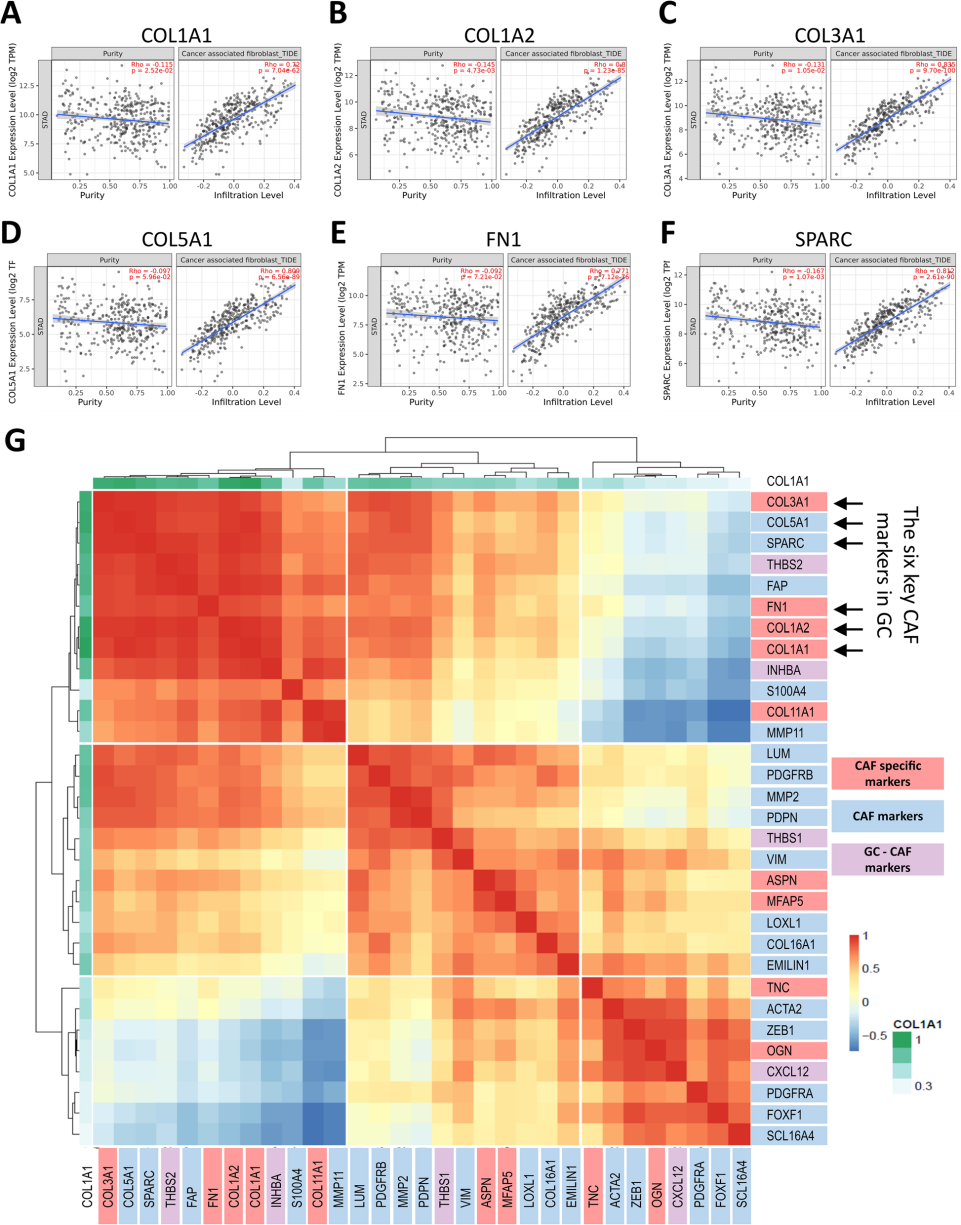
Correlation of the six key CAF markers with the CAF infiltration and other CAF markers. The correlation of **A ***COL1A1*, **B ***COL1A2*, **C ***COL3A1, ***D ***COL5A1*, **E ***FN1*, **F ***SPARC* with CAF infiltration in gastric cancer (TIDE algorithm in TIMER2.0. was used to infer the CAF infiltration levels in TCGA stomach adenocarcinoma samples). **G** Correlation heatmap for the six key CAF markers in gastric cancer (GC) with an extended list of other CAF markers. Correlation data was extracted from TIMER2.0 using the “gene correlation” module. Heatmap was created in imageGP using hierarchical clustering. The six key CAF markers in GC are shown with black arrows. Genes are classified as CAF-specific markers (red), CAF markers (blue), and GC-CAF markers (CAF markers identified in GC) (lilac)

To validate the six key CAF markers we detected with a network-based analysis, we investigated their correlation with CAF infiltration and other CAF markers in gastric cancer. Correlation analysis in TIMER2.0 revealed a statistically significant correlation for all six CAF markers with CAF infiltration in the TCGA stomach adenocarcinoma dataset (Fig. 4a-f). The correlation of these markers with the CAF infiltration was even higher than that of poor prognostic CAF signature genes recently identified in gastric cancer: *THBS1, THBS2, INHBA* (inhibin A), *CXCL12* (C-X-C motif chemokine ligand 12)*, TGFB2* (transforming growth factor-beta 2), *VEGFB* (vascular endothelial growth factor B), *COL10A1* (collagen type X alpha 1 chain), *AREG* (amphiregulin) and *EFNA*5 (ephrin A5) (Additional file 2: Fig. S2) [8, 32, 33]. These findings validated the central role of *COL1A1, COL1A2, COL3A1, COL5A1*, and *FN1* as CAF markers in gastric cancer.

Then we investigated the correlation of the six key CAF markers with the total list of CAF markers at the gene expression level in TCGA stomach adenocarcinoma samples (Fig. 4g). We also added *CXCL12, INHBA, THBS1*, and *THBS2* to the correlation analysis since they are suggested as CAF markers associated with an aggressive phenotype in gastric cancer [32–34]. In hierarchical clustering analysis of the correlation matrix, the six CAF markers were highly correlated and clustered with *COL11A1* (collagen type XI alpha1 chain), *FAP* (fibroblast activation protein), *INHBA, MMP11* (matrix metalloproteinase 11), *S100A4* (S100 calcium-binding protein A4) and *THBS2* (Fig. 4g).

### Verifying the differential expression and prognostic impact of the six CAF markers in gastric cancer

**Fig. 5:**
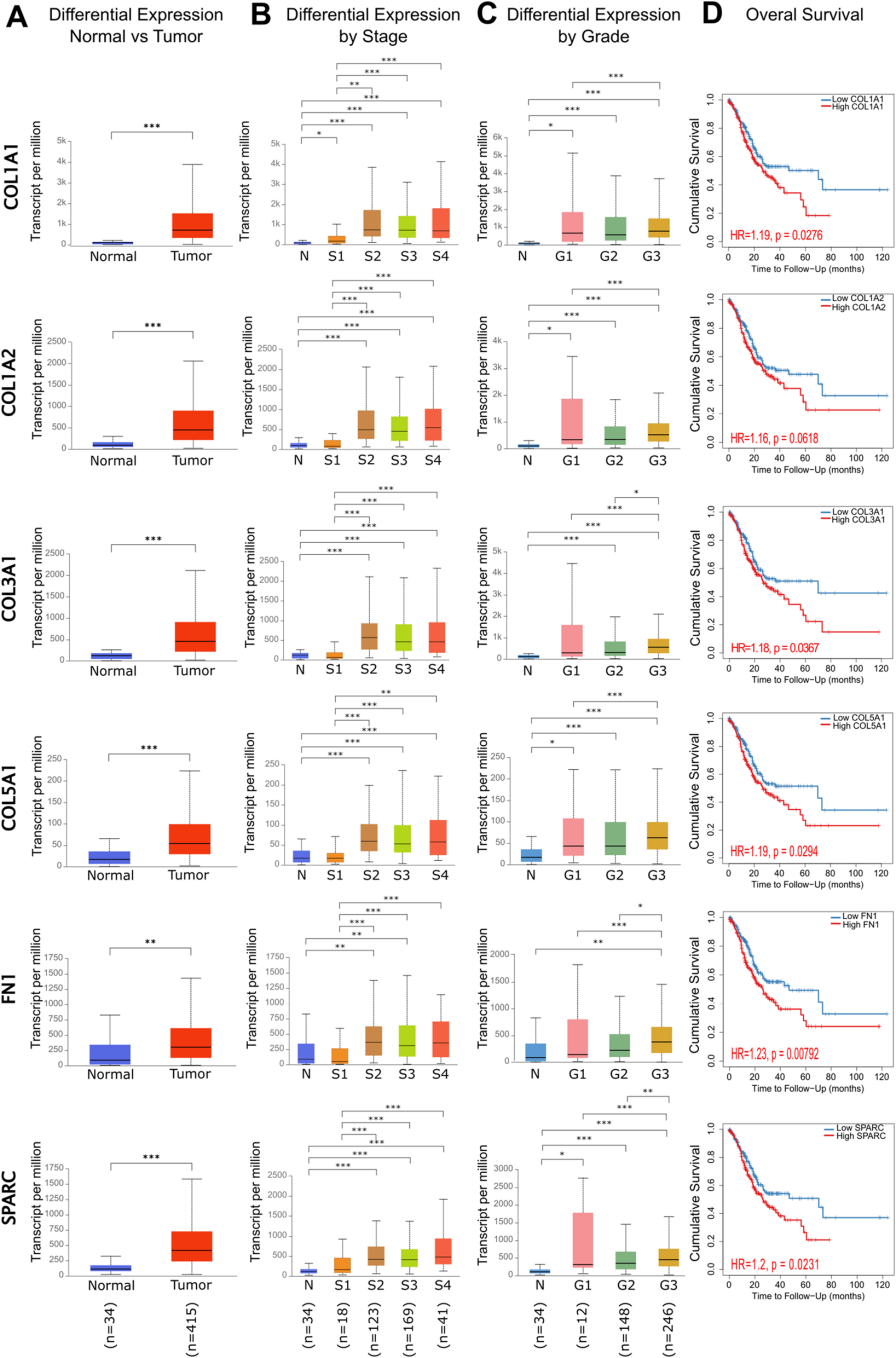
Differential expression and prognostic significance of the six key CAF markers in gastric cancer. **A** Differential expression of the CAF markers in normal tissues vs. primary stomach adenocarcinoma tissues. **B** Differential expression of the CAF markers by tumor stage in primary stomach adenocarcinoma (N: normal tissue, S1: stage1, S2: stage2, S3: stage3, S4: stage4). **C** Differential expression of CAF markers by tumor grade in stomach adenocarcinoma (N: normal tissue, G1:grade1, G2: grade2, G3: grade3). **D** Kaplan-Meier survival analysis of CAF markers in stomach adenocarcinoma. TCGA data which include 34 normal gastric tissues, and 415 primary stomach adenocarcinoma tissues were used for analysis (* *p* < 0.05, ** *p* < 0.01, *** *p* < 0.001). **A-C** Analysis was performed on the UALCAN. The numbers of samples in each group in the TCGA dataset are given in parenthesis at the bottom of the figure. **D** Analysis was performed on TIMER 2.0

To validate the differential expression of the six CAF markers in gastric cancer, we analyzed the TCGA data in UALCAN. The expression of all six markers was significantly higher in stomach adenocarcinoma samples, compatible with our results (Fig. 5a). The significant upregulation of these markers in gastric cancer was also verified on GEPIA2 using gastric cancer data from the GTEx dataset (data not shown).

To investigate whether the six CAF markers are involved in gastric cancer progression, we analyzed their differential expression by tumor stage and grade on UALCAN using the TCGA data. For *COL1A2, COL3A1, COL5A1, FN1*, and *SPARC*, there was no significant upregulation in stage 1 patients compared to healthy controls (Fig. 5b). However, their expression was significantly higher in stage 2, 3, and 4 samples than in the stage 1 samples. Only for *COL1A1*, the expression was high starting from stage 1. After stage 2, the increase in *COL1A1* expression became much more prominent. These findings suggest that *COL1A1* may be involved in both tumorigenesis and tumor progression. On the other hand, *COL1A2, COL3A1, COL5A1, FN1*, and *SPARC* may be more involved in tumor progression from stage 1 to stage 2, at which the tumor cells gain the ability to invade surrounding tissues.

The expression of *COL1A1, COL1A2, COL5A1*, and *SPARC* was significantly high starting from grade 1 (Fig. 5c). The medians of expression for these markers gradually increased in grade 2 and 3 samples. For *COL3A1* and *FN1*, the upregulation in grade 1 disease compared to healthy gastric tissue was not statistically significant due to the high variance in grade 1. However, their expression was significantly higher in grade 2 and grade 3 compared to grade 1 disease and normal tissue. These findings suggest that all six CAF markers may be associated with a poorly differentiated phenotype in gastric cancer.

Then we investigated the impact of upregulated *COL1A1, COL1A2, COL3A1, COL5A1, FN1*, and *SPARC* on patient survival with KM-survival analysis (Fig. 5d). High expression of *COL1A1, COL3A1, COL5A1, FN1*, and *SPARC* was significantly associated with poor survival in gastric cancer patients (*p* < 0.05). Although the survival curves for *COL1A2* high vs. low expression samples were different, the difference was not significant enough to suggest that the high expression of *COL1A2* is associated with poor survival (p = 0.0618). Despite that, the KM-survival curves for *COL1A2* high vs. low/medium expression samples from the TCGA database were statistically different (p = 0.029) (Additional file 2: Fig. S3). These findings support that *COL1A1, COL1A2, COL3A1, COL5A1, FN1*, and *SPARC* are associated with poor prognosis in gastric cancer.

### The differential expression of the six CAF markers in gastric cancer subtypes

**Fig. 6:**
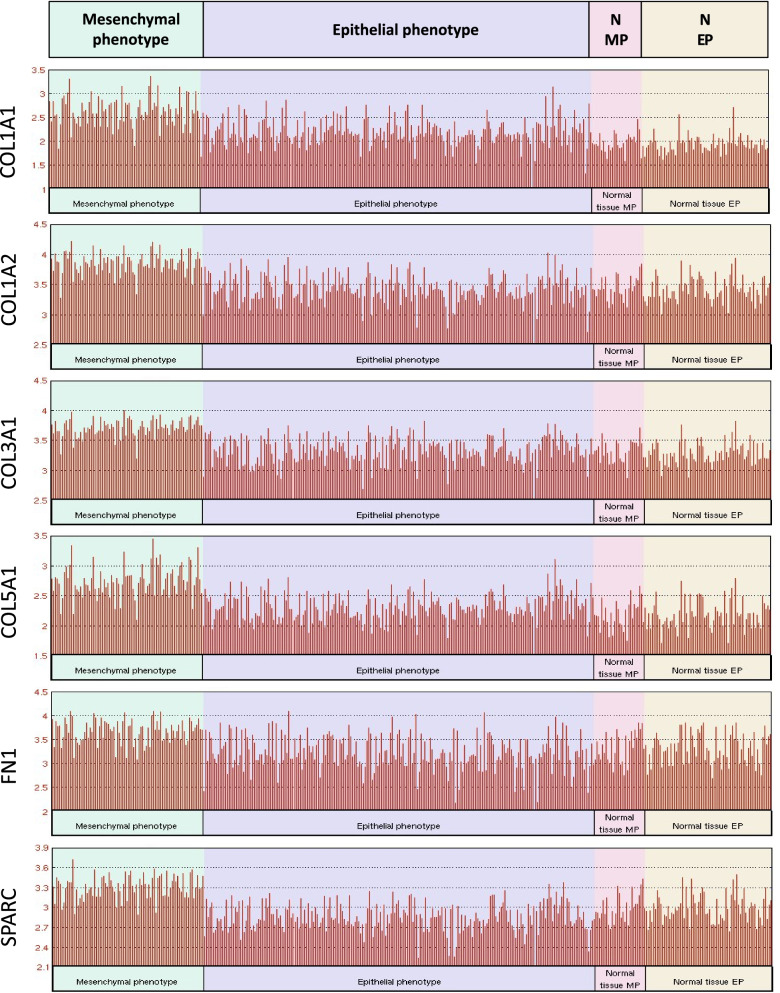
The differential expression of six CAF markers in gastric cancers with mesenchymal vs. epithelial phenotype. The differential expression of *COL1A1*, *COL1A2*, *COL3A1*, *COL5A1*, *FN1*, and *SPARC* in gastric cancers with mesenchymal vs. epithelial phenotype and normal gastric tissues from corresponding patients (abbreviated as N-MP: “Normal tissue-MP” for patients with mesenchymal phenotype gastric cancer and N-EP: “Normal tissue-EP” for patients with epithelial phenotype gastric cancer) in the Asian Cancer Research Group gastric cancer dataset (GSE66229). Analysis was performed on GEO2R

Gastric adenocarcinoma is a heterogeneous disease with two main histopathological subtypes: the intestinal-type and diffuse-type gastric adenocarcinoma. The intestinal type is characterized by organized glandular structures and responds better to chemotherapy. On the other hand, diffuse type is characterized by an undifferentiated phenotype and its response to chemotherapy is dismal [35]. Recent studies for the molecular characterization of gastric tumors also demonstrated that gastric tumors with a mesenchymal phenotype have a worse prognosis and poor response to chemotherapy compared to that with an epithelial phenotype [25]. Since CAF infiltration is associated with EMT in several cancers and CAFs can develop from mesenchymal cells [36–38], we asked whether the six key CAF markers are more dominant in gastric tumors with a mesenchymal phenotype. Our analysis in the ACGR cohort revealed that expression of all the six CAF markers was higher in gastric tumors with a mesenchymal phenotype compared with that of epithelial phenotype (Fig. 6). However, the expression profile of these markers did not significantly differ between the diffuse vs. intestinal subtypes (Additional file 2: Fig. S4). Although diffuse-type gastric adenocarcinoma is more associated with a mesenchymal phenotype compared with the intestinal-type [35], it exhibits inter-patient variability in terms of the mesenchymal markers [25]. Therefore, mesenchymal phenotype seems to be a better indicator for the expression of six CAF markers, hence the CAF infiltration.

### Pan-cancer analysis of the six CAF markers at the TCGA database

**Fig. 7:**
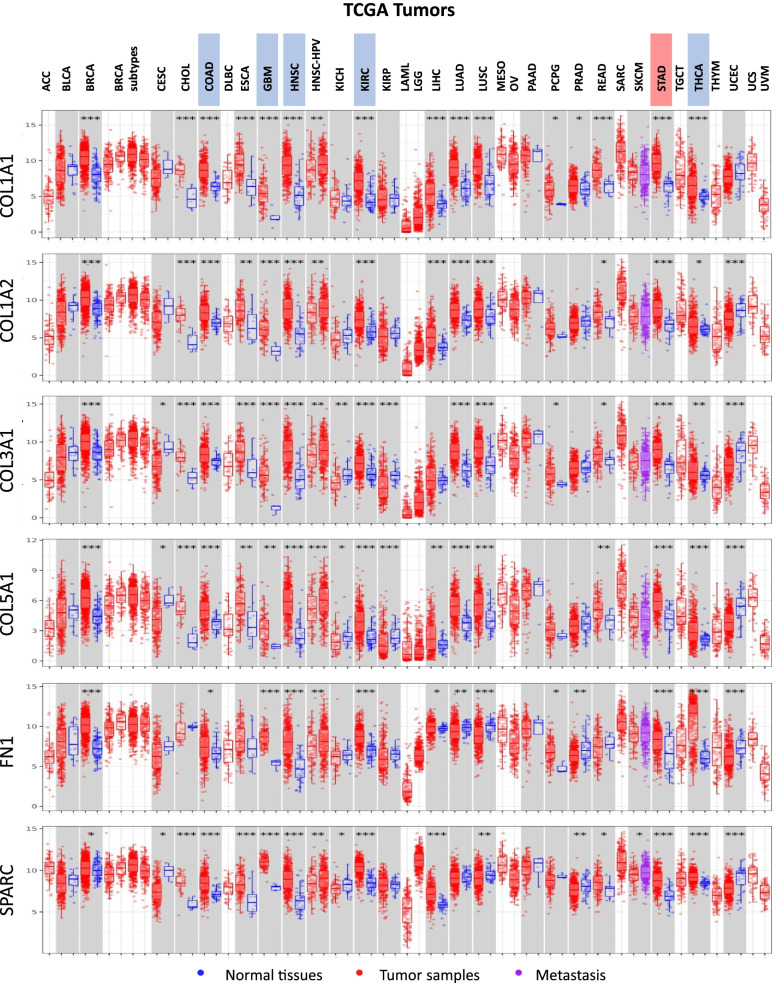
Pan-cancer expression profiles of the six key CAF markers in the TCGA dataset. Pan-cancer differential expression profiles of *COL1A1*, *COL1A2*, *COL3A1*, *COL5A1*, *FN1*, and *SPARC* in the TCGA dataset (* *p* < 0.05, ** *p* < 0.01, *** *p* < 0.001). Analysis was performed on TIMER 2.0. Stomach adenocarcinoma (STAD) is highlighted red and five other cancers (COAD: colon adenocarcinoma, GBM: glioblastoma, HNSC: head and neck squamous carcinoma, KIRC: kidney renal cell carcinoma, and THCA: thyroid carcinoma) at which all the six CAF markers were upregulated are highlighted blue in the legends. For a full list of TCGA cancer type abbreviations please refer to https://gdc.cancer.gov/resources-tcga-users/tcga-code-tables/tcga-study-abbreviations

**Fig. 8:**
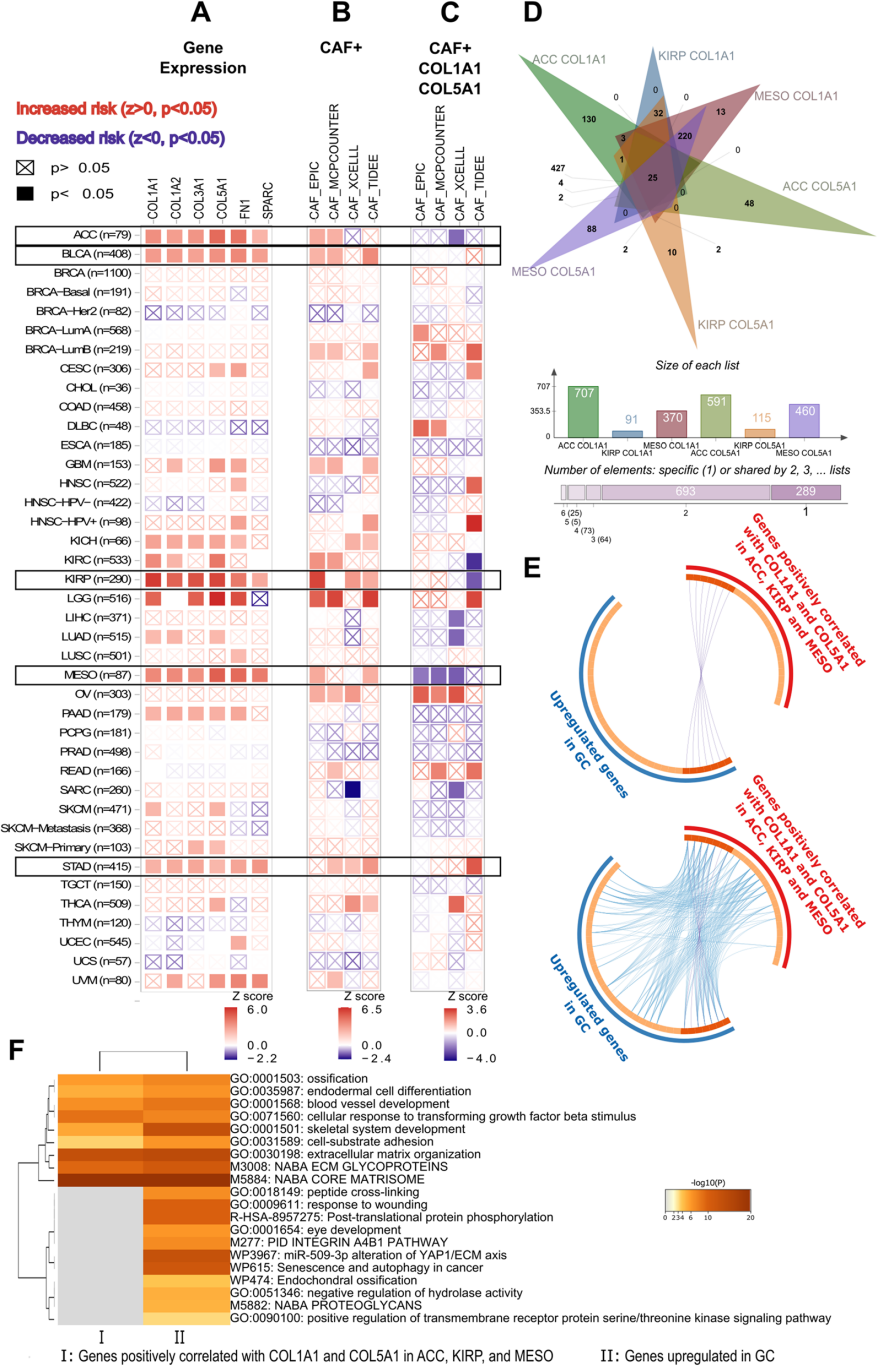
Prognostic impact of the six key CAF markers and the CAF infiltration in TCGA cancers. Heatmap of risk scores for **A** the expression of *COL1A1, COL1A2, COL3A1, COL5A1, FN1*, and *SPARC*, **B** the cancer-associated fibroblast (CAF) infiltration, **C** the expression of *COL1A1* and *COL5A1* plus CAF infiltration in different cancers at TCGA dataset. **A-C** z-score > 0 (*p* < 0.05) indicates increased risk and z-score < 0 (*p* < 0.05) indicates decreased risk. ACC: adrenocortical carcinoma, BLCA: bladder urothelial carcinoma, KIRP: kidney renal papillary cell carcinoma, MESO: mesothelioma, and STAD: stomach adenocarcinoma. Data was extracted from TIMER 2.0. **D** Venn analysis of genes highly correlated (r ≥ 0.5) with *COL1A1* and *COL5A1* in TCGA samples of ACC, KIRP, and MESO. **E** The circos plots that show how genes from the input gene lists- 25 common genes detected by Venn analysis at **(D)** (red outer circle) vs. 38 upregulated genes in gastric cancer (blue outer circle)- overlap. The dark orange color at the inner circle represents the genes that appear in both lists and the light orange color represents genes that are unique to that gene list. Purple lines (upper circos plot) link the same genes that are shared by the two lists. Blue lines (lower circos plot) link the different genes which fall into the same ontology term. **F** Enriched ontology clusters (GO/KEGG terms, canonical pathways) for 25 common genes correlated with *COL1A1* and *COL5A1* in ACC, KIRP, and MESO (I) vs. 38 upregulated genes in GC (II). **E-F** Analysis was performed on Metascape.org. For a full list of TCGA cancer type abbreviations please refer to https://gdc.cancer.gov/resources-tcga-users/tcga-code-tables/tcga-study-abbreviations

CAFs are the predominant cellular components in the microenvironment of various tumors, especially with a high stroma [39]. To understand whether the six CAF markers we identified in gastric cancer are also upregulated in other cancers we performed a pan-cancer analysis of these markers in the TCGA dataset. Although each of the markers was upregulated in more than half of the cancers, there were five cancer types besides stomach adenocarcinoma in which all the six markers were upregulated: colon adenocarcinoma (COAD), glioblastoma (GBM), head and neck squamous carcinoma (HNSC), kidney renal cell carcinoma (KIRC) and thyroid carcinoma (THCA) (Fig. 7). Despite that, high expression of all the six markers was not associated with poor prognosis in these cancers (Fig. 8a). CAF infiltration was not even associated with a poor prognosis in colon adenocarcinoma and head and neck squamous carcinoma among these cancers in the TCGA dataset (Fig. 8b).

CAFs display a heterogenous gene expression profile in the stroma of distinct cancers. Hence, they play anti-tumor or tumor-promoting roles depending on the tumor type [7]. Although the six CAF markers were upregulated in colon adenocarcinoma, glioblastoma, head and neck squamous carcinoma, kidney renal cell carcinoma, and thyroid carcinoma, the overall CAF secretome or inner cellular machinery that responds to the six CAF markers may not end up with a poor prognostic response in these tumors. Therefore, the six CAF markers we identified in gastric cancer may not necessarily indicate a poor prognostic impact on CAF infiltration in these tumors. With further analysis, we identified four other cancers in which all six markers and CAF infiltration were associated with poor prognosis: adrenocortical carcinoma (ACC), bladder urothelial carcinoma (BLCA), kidney renal papillary cell carcinoma (KIRP), and mesothelioma (MESO) (Fig. 8a). We comparatively analyzed these cancers with gastric cancer to establish a poor prognostic CAF gene signature with high specificity to gastric cancer.

### Prognostic impact of the six CAF markers on gastric tumors with CAF infiltration

CAF infiltration by itself is associated with a hazard ratio of 5.24 in stomach adenocarcinoma in the KM-survival analysis of TCGA data (Table 3). Moreover, the outcome of CAF infiltration worsens with the increasing tumor stage in gastric cancer (Additional file 2: Fig. S5). We investigated whether high expression of the six CAF markers further worsens the prognosis in gastric tumors with CAF infiltration. We performed Cox proportional hazard regression analysis that considers both gene expression profiles and CAF infiltration in the TCGA stomach adenocarcinoma samples.

**Table 3:** Parameters of the multivariate Cox proportional regression model for six key CAF markers and CAFs. TCGA data for stomach adenocarcinoma samples were analyzed in the TIMER2.0 immune association - outcome module using the TIDE algorithm for the allocation of samples to the high vs. low CAF infiltration groups. Likelihood ratio and Score log-rank tests were performed (CAF: cancer-associated fibroblast, CI: confidence interval, HR: hazard ratio)

Variate/s	HR of CAF infiltration	95% CI	z-score	***p***-value
**CAF**	5.240	2.004–13.702	3.377	0.001
**CAF, *COL1A1***	5.430	1.392–21.179	2.437	0.015
**CAF, *COL1A2***	5.631	1.218–26.033	2.212	0.027
**CAF, *COL3A1***	5.916	1.078–32.465	2.047	0.041
**CAF, *COL5A1***	8.584	1.767–41.695	2.666	0.008
**CAF, *FN1***	4.247	0.956–18.880	1.900	0.057
**CAF, *SPARC***	3.042	0.599–0.878	1.342	0.180
**CAF, *COL1A1, COL1A2***	5.789	1.164–28.802	2.145	0.032
**CAF, *COL1A1, COL3A1***	6.167	1.034–36.775	1.997	0.046
**CAF, *COL1A1, COL5A1***	11.654	2.247–60.438	2.924	0.003
**CAF, *COL1A2, COL3A1***	5.968	1.074–33.168	2.041	0.041
**CAF, *COL1A2, COL5A1***	7.701	1.550–38.253	2.496	0.013
**CAF, *COL3A1, COL5A1***	6.339	1.151–34.913	2.121	0.034
**CAF, *COL1A1, COL1A2, COL3A1, COL5A1***	10.176	1.532–67.610	2.401	0.016

Out of the six CAF markers, the expression of *COL1A1, COL1A2, COL3A1,* or *COL5A1* increased the z-score and hazards ratio for CAF infiltration (Table 3). The high *COL5A1* expression led to the highest increase in the risk for poor survival (z = 2.666, HR = 8.584). The high FN1 or SPARC expression did not increase the z-score and hazards ratio for CAF infiltration.

After that, we investigated whether the high expression of dual combinations of *COL1A1, COL1A2, COL3A1,* or *COL5A1* exacerbates the outcome of CAF infiltration in stomach adenocarcinoma (Table 3). Concomitant high expression of *COL1A1* and *COL5A1* increased the hazard ratio most for CAF infiltration in TCGA samples (z = 2.924, HR = 11.654). The hazard ratio of CAF infiltration with this dual gene combination was even higher than that with the quadruple combination of collagen subunits. We also investigated the impact of CAF markers highly clustered with *COL1A1* and *COL5A1* in correlation analysis, namely *THBS2, FAP, INHBA, S100A4, COL11A1*, or *MMP11,* on the outcome of CAF infiltration in stomach adenocarcinoma. Except for *MMP11*, all slightly increased the hazard ratio for CAF infiltration (Additional file 1: Table S7).

Recently, Liu et al. suggested *TGFB2, VEGFB, COL10A1, AREG* and *EFNA5*; and Grunberg et al. suggested *THBS1*, *THBS2,* and *INHBA* as poor prognostic signatures for CAF infiltration in gastric cancer [8, 32]. To compare the prognostic significance of these two gene signatures with that of *COL1A1* and *COL5A1,* we investigated the Cox regression models for these two signatures. The z-scores and hazard ratios for both signatures were lower than those for *COL1A1* and *COL5A1* (Additional file 1: Table S8). These findings suggested a high potential for *COL1A1* and *COL5A1* as a poor prognostic signature in CAF infiltrated gastric tumors.

### Prognostic significance of COL1A1 and COL5A1 for CAF infiltration in other cancers

At the next step, we asked whether *COL1A1* and *COL5A1* increase the poor prognostic impact of CAF infiltration in four cancers (adrenocortical carcinoma, bladder urothelial carcinoma, kidney renal papillary cell carcinoma, and mesothelioma) with a poor outcome profile for the six CAF markers and CAF infiltration like gastric cancer. Interestingly, the addition of *COL1A1* and *COL5A1* to the CAF Cox model led to a decreased risk score for CAF infiltration in adrenocortical carcinoma, kidney renal papillary cell carcinoma, and mesothelioma (Fig. 8c).

**Fig. 9:**
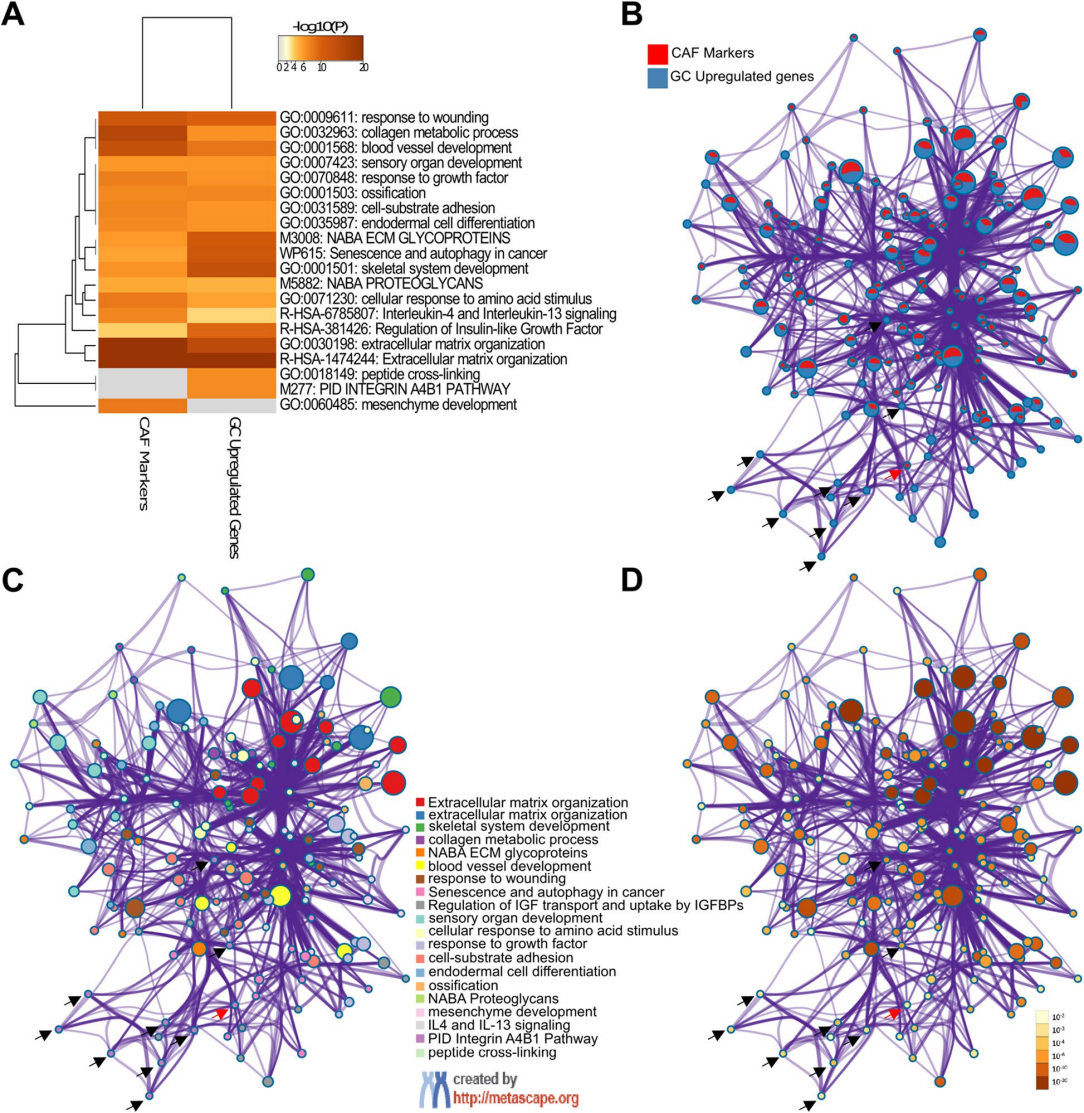
Enriched ontology clusters for the cancer-associated fibroblast markers and upregulated genes in gastric cancer. **A** Dendrogram of the enriched ontology clusters (GO/KEGG terms, canonical pathways) for the CAF markers and upregulated genes in GC. **B** Network representation of enriched terms for a combined list of the CAF markers and upregulated genes in gastric cancer (GC). Each term is represented by a circle node, where its size is proportional to the number of the input genes that fall into that term. The nodes are presented as pie sectors where each pie sector is proportional to the number of hits originating from the CAF markers list (red) and upregulated genes in GC (blue). **C** The gene ontology terms for the same network nodes in B where each color represents different cluster identities given in the label. **D** Representation of the same network nodes in B and C colored by *p*-value, as shown in the color scale. **B-D** The black arrows show the nodes that fall into the Integrin α4β1 pathway in GC upregulated genes list. Only one node that falls into the Integrin α4β1 pathway shown with the red arrow was common in both the CAF markers list and the GC upregulated genes list. The data was analyzed, and the network layouts were prepared in Metascape.org

Identifying the players for the opposing roles of *COL1A1* and *COL5A1* in different cancers could reveal new insights into the field. To predict possible players, we extracted the list of genes that correlate with the expression of *COL1A1* and *COL5A1* in adrenocortical carcinoma, kidney renal papillary cell carcinoma, and mesothelioma. We identified 25 genes that are highly correlated (rho ≥ 0.5) with both *COL1A1* and *COL5A1* in all three cancers (Fig. 8d). Then we comparatively analyzed this list with the list of genes upregulated in gastric cancer. The two lists shared seven genes (Fig. 8e, upper circos plot). The 18 genes common to the three cancers fell into the same gene ontology as the 27 genes upregulated in gastric cancer (Fig. 8e, lower circos plot). Despite these overlaps, more than ten ontologies are differentially enriched in the list of upregulated genes in gastric cancer (Fig. 8f). Among these, “integrin α_4_β_1_ pathway” and “peptide crosslinking” were striking, since they were the two ontologies that are also enriched at the upregulated gene list in gastric cancer compared to the CAF markers list (Fig. 9a). Network layouts for enriched ontology clusters given in Fig. 9b-d better demonstrate that the most striking difference for upregulated genes in gastric cancer was the enrichment of the “integrin α_4_β_1_ pathway” compared with the CAF markers list. These observations emphasized the role of the “integrin α_4_β_1_ pathway” together with CAF infiltration in gastric cancer and proposed the integrin α_4_β_1_ signaling as a poor prognostic factor for CAF infiltration specifically in gastric cancer.

### Contribution of Integrin α4β1 pathway to the poor prognostic impact of COL1A1, COL5A1, and CAF infiltration in stomach adenocarcinoma

Integrins are heterodimeric transmembrane proteins that are involved in cell-cell or cell-ECM adhesions. They bind ECM components, mainly collagen, and fibronectin, activate intracellular signaling pathways, and regulate cell survival, proliferation, migration, and differentiation. Integrin α_4_β_1_ is a heterodimer of integrin α_4_ (ITGA4) and integrin β_1_ (ITGB1). ITGB1 couples with a large variety of α integrin subunits [40]. However, ITGA4 couples with integrin β_1_ or β_7_ subunits [41, 42].

The integrin α_4_β_1_, also known as very late antigen-4 (VLA-4), is expressed on various immune cells, mediating the migration of leukocytes to the inflammatory sites via interaction with VCAM-1 (vascular cell adhesion protein 1) [41]. Additionally, it binds to ECM components and takes part in fibronectin assembly [43]. Increased expression of α_4_β_1_ integrin is associated with tumor progression and chemoresistance in cancer. The interaction of integrin α_4_β_1_ on the tumor cell membrane with the VCAM-1 on vascular endothelial cells is involved in metastasis [41]. The interaction of α_4_β_1_ integrin with fibronectin suppressed apoptosis via FAK-mediated suppression of p53, and PI3K/Akt mediated upregulation of Bcl-2 in myeloma cells. Increased integrin α_4_β_1_ expression was associated with increased binding of melanoma cells to collagen I and collagen IV, and invasion through fibronectin [44]. Moreover, α_4_ integrin was suggested to affect a drug efflux mechanism independent of its coupling with β_1_ integrin [45]. However, the mechanisms by which integrin α_4_β_1_ heterodimer or α_4_ integrin monomer contribute to invasion, metastasis, and chemoresistance in cancer are not exactly known.

To understand whether the integrin α_4_β_1_ potentiates the poor prognostic impact of CAFs, we analyzed the Cox regression model for CAF infiltration that considers the expression of *ITGA4, ITGB1,* or both in addition to *COL1A1* and *COL5A1.* The addition of *ITGA4* to the model increased the hazard ratio and z-score in stomach adenocarcinoma (z = 2.963, HR = 12.247) (Table 4). However, only *ITGB1* or *ITGA4* and *ITGB1* slightly decreased the hazard ratio and z-score, which may be due to a less selective coupling of ITGB1 with several integrin α subtypes (Additional file 1: Table S9). These findings supported that *ITGA4* may worsen the poor prognostic impact of CAFs in gastric cancer.

**Table 4:** Parameters of the stepwise multivariate Cox proportional regression model for cancer-associated fibroblasts and signature genes. TCGA data for stomach adenocarcinoma, adrenocortical carcinoma, kidney renal papillary cell carcinoma, and mesothelioma were analyzed in the TIMER2.0 immune association - outcome module using the TIDE algorithm for the allocation of samples to the high vs. low CAF infiltration groups. Likelihood ratio and Score log-rank tests were performed (CAF: cancer-associated fibroblast, CI: confidence interval, HR: hazard ratio)

Variate/s	HR of CAF infiltration	95% CI	z-score	***p***-value
	**Stomach Adenocarcinoma**			
**CAF, *COL1A1, COL5A1***	11.654	2.247–60.438	2.924	0.003
**CAF, *COL1A1, COL5A1, ITGA4***	12.247	2.335–64.240	2.963	0.003
**CAF, *COL1A1, COL5A1, ITGA4, EMILIN1***	28.315	4.143–193.524	3.409	0.001
**CAF, *COL1A1, COL5A1, ITGA4, EMILIN1, TSPAN9***	36.813	5.007–270.645	3.543	0.000
	**Adrenocortical Carcinoma**			
**CAF, *COL1A1, COL5A1***	0.011	0.000–10.094	−1.297	0.195
**CAF, *COL1A1, COL5A1, ITGA4***	0.011	0.000–11.744	−1.270	0.204
**CAF, *COL1A1, COL5A1, ITGA4, EMILIN1***	0.007	0.000–9.801	−1.341	0.180
**CAF, *COL1A1, COL5A1, ITGA4, EMILIN1, TSPAN9***	0.014	0.000–23.333	−1.126	0.260
	**Kidney Renal Papillary Cell Carcinoma**			
**CAF, *COL1A1, COL5A1***	0.002	0.000–0.223	−2.566	0.010
**CAF, *COL1A1, COL5A1, ITGA4***	0.002	0.000–0.241	−2.535	0.011
**CAF, *COL1A1, COL5A1, ITGA4, EMILIN1***	0.002	0.000–0.287	−2.427	0.015
**CAF, *COL1A1, COL5A1, ITGA4, EMILIN1, TSPAN9***	0.002	0.000–0.515	−2.197	0.028
	**Mesothelioma**			
**CAF, *COL1A1, COL5A1***	0.000	0.000–0.128	−2.513	0.012
**CAF, *COL1A1, COL5A1, ITGA4***	0.000	0.000–0.166	−2.443	0.015
**CAF, *COL1A1, COL5A1, ITGA4, EMILIN1***	0.000	0.000–0.737	−2.036	0.042
**CAF, *COL1A1, COL5A1, ITGA4, EMILIN1, TSPAN9***	0.001	0.000–0.133	−2.834	0.005

ITGA4 interacts with signaling molecules, receptors, and kinases that take part in ECM organization, integrin signaling, and cell-matrix adhesion (Additional file 2: Fig. S6A-B). Among the integrin α_4_β_1_ partners FN1, osteopontin (secreted phosphoprotein1: SPP1), THBS1, and EMILIN-1 (Elastin microfibril interface-located protein 1) are ECM proteins; JAM2 (junctional adhesion molecule 2), JAM3 (junctional adhesion molecule 3), MADCAM1 (mucosal vascular addressin cell adhesion molecule 1), and VCAM1 are membrane-bound proteins. Their interaction with integrin α4β1 makes them potential players for the poor prognostic impact of ITGA4 [43, 46–49]. We asked whether *FN1,* one of the six CAF markers we detected in network analysis, strengthens the poor prognostic effect of *ITGA4* on CAF infiltration. However, the addition of *FN1* to the *COL1A1*, *COL5A1,* and *ITG4* Cox regression model decreased the poor prognostic impact of CAF in stomach adenocarcinoma (Additional file 1: Table S10). *THBS1* acted similarly and decreased the hazard ratio. *JAM2, JAM3, MADCAM1*, *SPP1,* and *VCAM1* slightly increased the hazard ratio. On the other hand, *EMILIN1* substantially increased the poor prognostic impact of CAFs in the *COL1A1*, *COL5A1,* and *ITG4* model, raising the hazard ratio from 12.247 to 28.315 (Table 4).

The EMILIN-1 is a member of the elastin microfibrillar interface proteins (EMILINs) family, expressed as a homotrimer at the ECM [50]. Since fibroblasts are the major sources of EMILIN-1 at the ECM, it is accepted as a fibroblast marker [51]. The interaction of EMILIN-1 with integrin α_4_β_1_ is involved in cell adhesion and migration [52]. The increase in the poor prognostic impact of CAFs in stomach adenocarcinoma with the addition of *EMILIN1* as a covariate to the Cox model was surprising since EMILIN-1 is known as a tumor suppressor that exerts anti-proliferative action via integrin α_4_β_1_ in cell and in vivo models [50, 53]. Despite that, some reports suggest a pro-tumorigenic role for EMILIN-1 in ovarian serous tumors and osteosarcoma [54, 55].

**Fig. 10:**
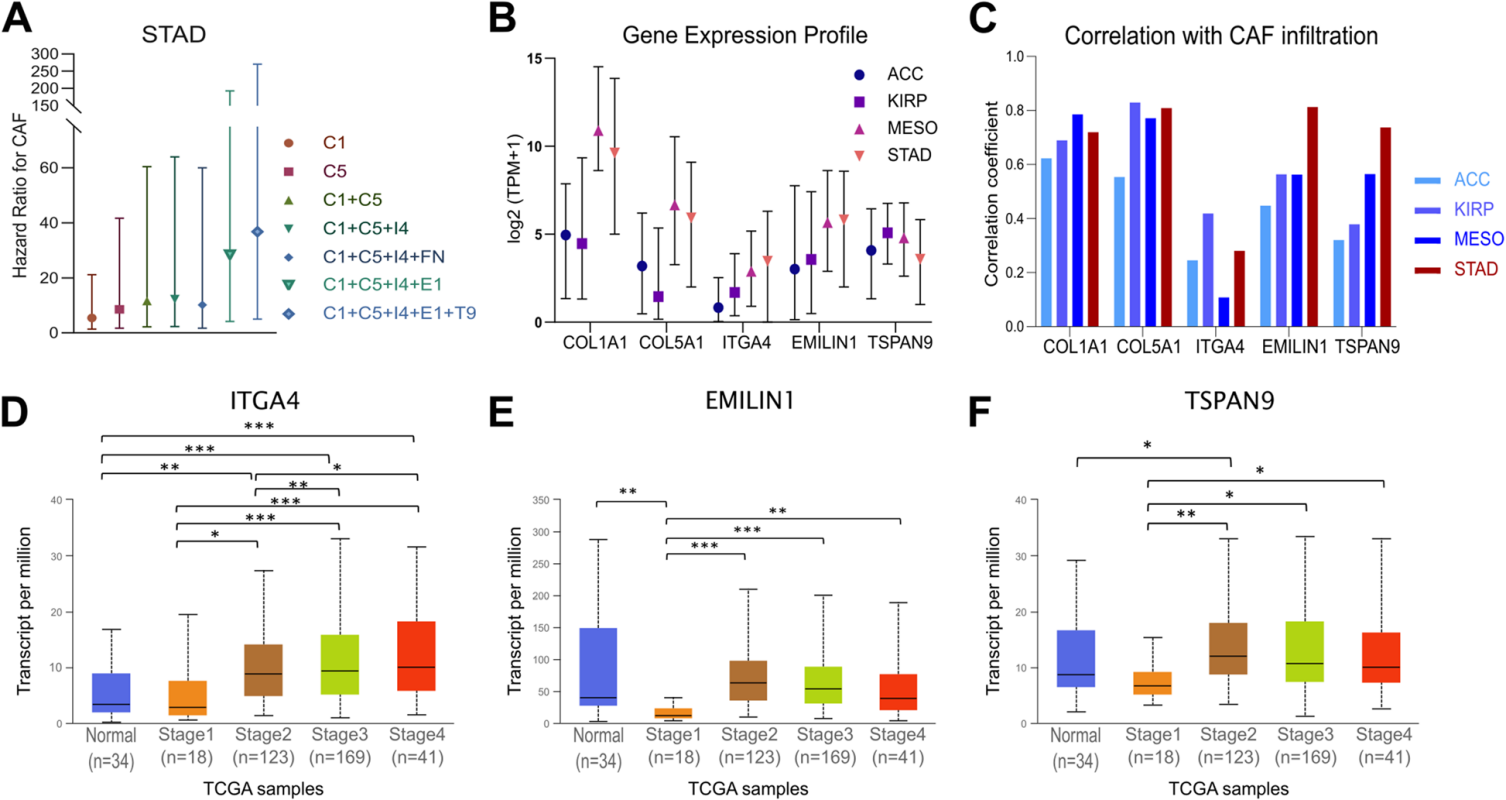
Prognostic impact and the expression profile of the cancer-associated fibroblast signature genes. **A** The hazard ratios of CAF infiltration for different gene expression profiles in STAD. C1: *COL1A1*, C5: *COL5A1*, I4: *ITGA4,* E1: *EMILIN1,* F1: *FN1* and T9: *TSPAN9.* Bars indicate 95% confidence intervals. Data was extracted from TIMER2.0. **B** Gene expression profile for *COL1A1*, *COL5A1*, *ITGA4, EMILIN1,* and *TSPAN9* in ACC, KIRP, MESO, and STAD. Symbols show the medians for transcripts per million (TPM) in each cancer at the TCGA dataset. Data was extracted from UALCAN. **C** Correlation between CAF infiltration and the expression of *COL1A1*, *COL5A1*, *ITGA4, EMILIN1,* and *TSPAN9* in ACC, KIRP, MESO, and STAD. Data was extracted from TIMER2.0. using the TIDE algorithm. Differential expression of **D ***ITGA4, ***E ***EMILIN1,* and **F ***TSPAN9* by tumor stage in primary STAD. The TCGA data, which includes 34 normal tissues and 415 primary stomach adenocarcinoma tissues, was used for analysis. Analysis was performed on UALCAN (unpaired t-test, * *p* < 0.05, ** *p* < 0.01, *** *p* < 0.001). (ACC: adrenocortical carcinoma, BLCA: bladder urothelial carcinoma, KIRP: kidney renal papillary cell carcinoma, MESO: mesothelioma, and STAD: stomach adenocarcinoma)

A recent study reported that the action of EMILIN-1 to inhibit the MAPK pathway and suppress proliferation in gastric cancer cells might depend on Tetraspanin9 (TSPAN9) [56], which is a member of the tetraspanin family membrane receptors with four transmembrane domains. These receptors are involved in signal transduction, cell adhesion, invasion, and migration. TSPAN9 is alluded to have anti-cancer effects in gastric cancer, suppressing proliferation, invasion, and migration in gastric cancer cell lines [57, 58]. Despite that, adding *TSPAN9* to the *COL1A1, COL5A1, ITGA4,* and *EMILIN1* Cox model further increased the hazard ratio to 36.813 for CAF infiltration in stomach adenocarcinoma samples (Fig. 10a, Table 4). However, the hazard ratios remained zero with the stepwise addition of *ITGA4, EMILIN1,* and *TSPAN9* to the Cox model in adrenocortical carcinoma, kidney renal papillary cell carcinoma, and mesothelioma (Table 4). All this data suggested *COL1A1*, *COL5A1*, *ITGA4, EMILIN1,* and *TSPAN9* as a poor prognostic CAF signature with high specificity to stomach adenocarcinoma.

We further investigated the expression profiles of the signature genes and their correlation with CAF infiltration (Fig. 10b-e). Mesothelioma and stomach adenocarcinoma displayed a higher expression profile for the *COL1A1*, *COL5A1*, *ITGA4,* and *EMILIN1*, in comparison to adrenocortical carcinoma and kidney renal papillary cell carcinoma. The expression of *TSPAN9* was similar in all four cancers (Fig. 10b). Correlation between the *COL1A1* or *COL5A1* expressions and CAF infiltration was strong in all four cancers (rho > 0.5). *ITGA4* expression poorly correlated with the CAF infiltration, but the correlation was slightly higher in kidney renal papillary cell carcinoma and stomach adenocarcinoma. Strikingly, the correlation of *EMILIN1* and *TSPAN9* with *CAF* infiltration was quite strong in stomach adenocarcinoma compared to a poorer correlation in the other three cancers (Fig. 10c).

Further investigation revealed that the expression of *ITGA4* increases with stage in stomach adenocarcinoma (Fig. 10d). Although there was a significant decrease in *EMILIN1* and *TSPAN9* levels in stage 1 compared to healthy stomach tissue, their expression increased again at stage 2, reaching the level of or above that of normal tissues (Fig. 10e-f). A similar pattern was not observed for adrenocortical carcinoma, kidney renal papillary cell carcinoma, and mesothelioma (Additional file 2: Fig. S7).

The KM-survival analysis did not indicate a prognostic role for *ITGA4, EMILIN1*, or *TSPAN9* in stomach adenocarcinoma per se (Additional file 2: Fig. S8A-C). However, their hazard ratios increased with the stage (Additional file 2: Fig. S8D-F). This was in parallel to the increase in the poor prognostic impact of CAF infiltration by stage in stomach adenocarcinoma (Additional file 2: Fig. S5), suggesting a stage and CAF dependent role for *ITGA4, EMILIN1*, and *TSPAN9.*

### Search on drugs that target the poor prognostic CAF signature

**Fig. 11:**
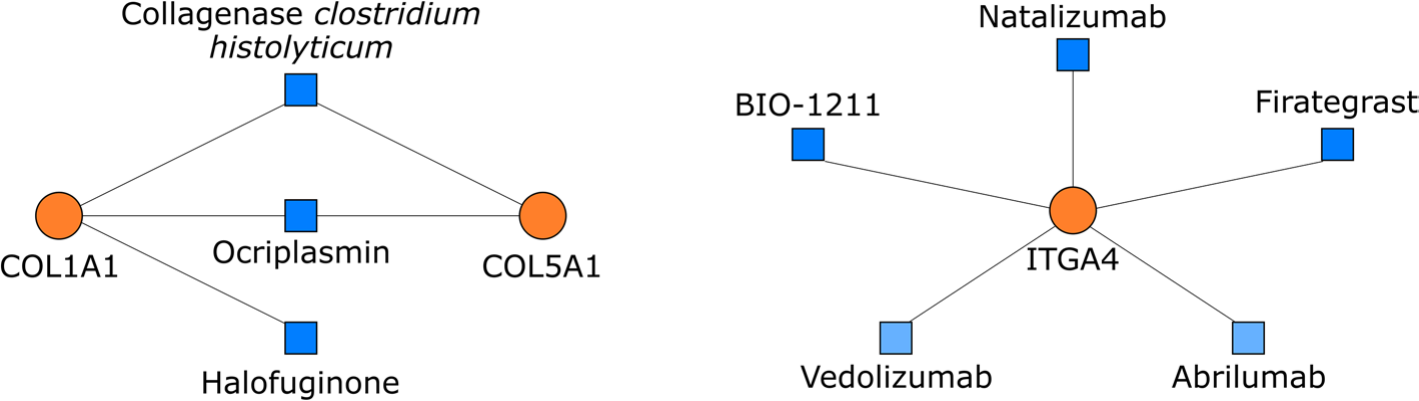
Currently available drugs that target COL1A1, COL5A1, and ITGA4. Drugs (shown with blue squares) listed in DrugBank and Drug-gene interaction database that target COL1A1, COL5A1, or ITGA4 (shown with orange circles). Among the ITGA4 targeting drugs, the action of abrilumab, and vedolizumab, which are shown in light blue, are specific to integrin α4β7 rather than integrin α4β1. Natalizumab, firategrast, and BIO-1211 target integrin α4

Lastly, we searched for currently available drugs that target the five signature genes *COL1A1, COL5A1, ITGA4, EMILIN1,* and *TSPAN9*. The drugs that target *EMILIN1,* and *TSPAN9* are not currently available. But our search on DrugBank and DGIB revealed three agents which target COL1A1 and COL5A1: collagenase *clostridium histolyticum*, halofuginone, and ocriplasmin; and three agents which target ITGA4: natalizumab, firategrast, and BIO-1211 (Fig. 11).

Collagenase *clostridium histolyticum* and ocriplasmin are enzymes that cleave COL1A1 and COL5A1. They also have proteolytic activity on COL3A1 and FN1, respectively [59, 60]. Collagenase *clostridium histolyticum* is used on skin ulcers to hasten wound healing and Dupuytrens’ disease to resolve contractures by digesting collagen [61, 62]. Intra-tumoral or intravenous injection of collagenase increased the diffusion of large drug molecules in tumor models [63]. Intraperitoneal administration of collagenase was reported to increase the efficacy of chemotherapy by cleaving the tumor stroma in a rat model of colorectal cancer peritoneal metastasis [64]. Ocriplasmin is used to remove adhesions in symptomatic vitreomacular adhesion [60]. Like our study, another bioinformatics study suggested ocriplasmin as a potential anti-cancer agent [65]. But, to the best of our knowledge, ocriplasmin has not been tested in cancer before.

Halofuginone is an alkaloid that suppresses the expression of the COL1A1 gene, cell migration, and ECM formation. Besides its’ antifibrotic and anti-angiogenetic actions, halofuginone shows antiproliferative effects by inhibiting TGFβ/Smad3 signaling [66, 67]. Halofuginone, showed an apoptotic effect in prostate cancer and Wilms’ tumor cells by inhibiting the transformation of fibroblasts to myofibroblasts [68], which carry similar features to CAFs [7]. Halofuginone also acted synergistically with gemcitabine and suppressed tumorigenesis in a mouse pancreatic cancer model by reducing the number of stromal myofibroblasts and generation of ECM [69].

Integrin α_4_β_1_ is a significant therapeutic target in chronic inflammatory diseases and cancer. Natalizumab, the monoclonal antibody against integrin α_4_ subtype, was approved in multiple sclerosis and inflammatory bowel disease. However, its long-term use is associated with progressive multifocal leukoencephalopathy [49]. Although abrilumab and vedolizumab are listed as integrin α_4_ targeting agents, their action is specific to integrin α_4_β_7_ heterodimer [70, 71]. Besides monoclonal antibodies, small molecule inhibitors that target integrin α_4_ such as firategrast and BIO-1211 are available [72, 73]. To the best of our knowledge, these agents have not been tested for their therapeutic efficacy in cancer yet.

## Discussion

In this study, we performed a comprehensive bioinformatic analysis to identify poor prognostic CAF markers targeting which may have a therapeutic potential in gastric cancer patients. Our network-based approach revealed an upregulated ECM protein hub where the CAF markers *COL1A1, COL1A2, COL3A1, COL5A1, FN1*, and *SPARC* were the most central genes. High expression of all these CAF markers was associated with high CAF infiltration, tumor progression, mesenchymal phenotype, and decreased survival in gastric cancer patients. Based on the comparative pan-cancer analysis of these key CAF markers and a comprehensive literature search we identified *COL1A1*, *COL5A1*, *ITGA4, EMILIN1,* and *TSPAN9* as the signature genes, which potentiated the poor prognostic impact of CAF infiltration specifically in stomach adenocarcinoma. Our findings emphasize the key role of the tumor microenvironment and CAFs in gastric cancer.

Tumor cells dynamically interact with their microenvironment which consists of an ECM compartment and a cellular compartment. CAFs are pivotal cellular components in the tumor microenvironment. They secrete numerous ECM proteins, mainly fibrous collagens (type I, III, and V collagens) and fibronectin. They also remodel the ECM through matrix metalloproteinases (MMPs) which cleave the ECM components, and the lysyl oxidase (LOX) family enzymes which crosslink the collagens. The dynamic remodeling of ECM by CAFs facilitates cancer cell migration and invasion [7, 29]. Additionally, CAFs induce ECM stiffness in the tumor microenvironment, which is associated with chemoresistance and poor survival in many cancers [7, 29].

CAFs are mostly renowned for their pro-tumorigenic role. However, they can also exhibit an anti-tumorigenic role in a tumor-dependent manner. Whether they exhibit a pro-tumorigenic or an anti-tumorigenic role is highly determined by their secretome [74]. Therefore, identifying the poor prognostic signature of CAFs is of critical importance to develop anti-CAF strategies and select the patient populations that will benefit from these modalities. Our study and others’ findings demonstrated a poor prognostic role for CAFs in gastric cancer [8, 9] (Table 3 and Additional file 2: Fig. S5). Zeng et al. computationally analyzed the cell infiltration pattern in the tumor microenvironment of 1524 gastric cancer patients. The fibroblast infiltration was the greatest risk factor in tumor microenvironment phenotype with the poorest overall survival [10]. Despite that, the poor prognostic signature for CAFs is not clear in gastric cancer, and drugs that target CAF-specific pro-tumorigenic processes are not available in the clinic. Hence, in this study, we aimed to address these points.

To identify a poor prognostic signature for CAF infiltration in gastric cancer, we first identified the key CAF markers with a network-based approach in gastric cancer. We identified ECM components *COL1A1, COL1A2, COL3A1, COL5A1, FN1*, and *SPARC* as the pivotal CAF markers in gastric cancer, which were separately reported as biomarkers of gastric cancer in different studies [75–78]. We further validated their differential expression and poor prognostic significance in gastric cancer by analyzing the TCGA, GTEx, and ACRG cohorts. Additionally, we demonstrated that the high expression of these six CAF markers is associated with mesenchymal phenotype gastric cancer, which has a poorer prognosis and response to chemotherapy than the gastric tumors with the epithelial phenotype (Fig. 6). The higher content of stroma in mesenchymal phenotype gastric cancers and the role of EMT in CAF generation may explain this association. Interestingly, we could not observe a similar association in the diffuse histopathological subtype of gastric cancer which is more related to a mesenchymal phenotype compared with the intestinal histopathological type. However, Oh. Et al. suggested that the mesenchymal phenotype gastric cancers are a subset of diffuse gastric cancers [25]. Hence the heterogeneity of diffuse gastric cancers in terms of a mesenchymal or epithelial phenotype may explain this discrepancy.

In the next step, we performed pan-cancer profiling of the six key CAF markers. Although we identified five different cancers in which all the six CAF markers were upregulated, these markers or CAF infiltration did not exhibit poor prognostic effect in these cancers. These observations suggested that the six CAF markers we identified may have more critical roles in the poor prognostic impact of CAFs in gastric cancer. Then, we searched for other cancers in which all the six CAF markers and CAF infiltration are poor prognostic. We established a poor prognostic gene signature based on the comparative analysis of gastric cancer with these tumors, namely adrenocortical carcinoma, kidney renal papillary cell carcinoma, and mesothelioma.

Among the six CAF markers, we identified *COL1A1* and *COL5A1* as the two markers which potentiated the poor prognostic impact of CAF infiltration most in gastric cancer (Table 3). The opposite effect of this dual gene signature in adrenocortical carcinoma, kidney renal papillary cell carcinoma, and mesothelioma (Fig. 8e) led us to identify *ITGA4, EMILIN11*, and *TSPAN9* as interactors that potentiate the poor prognostic effect of CAFs specifically in gastric cancer. To the best of our knowledge, this is the first time the *ITGA4, EMILIN1*, and *TSPAN9* are put forth as poor prognostic signature genes for CAF infiltration in gastric cancer.

Recently, Liu et al. suggested *TGFB2*, *VEGFB*, *COL10A1*, *AREG*, and *EFNA5*; and Grunberg et al. suggested *THBS1*, *THBS2,* and *INHBA* as poor prognostic signatures for CAF infiltration in gastric cancer [8, 32]. But the z-score and hazard ratio for the dual *COL1A1* and *COL5A1* gene signature we identified was higher compared to both signatures (Table 4, Additional file 1: Fig. S8). The hazard ratio of CAF infiltration in *COL1A1*, *COL5A1, ITGA4*, *EMILIN1,* and *TSPAN9* signature was even higher (Table 4), presenting a 36.8 times higher risk of death in gastric tumors with high CAF infiltration. One possible advantage of *THBS1*, *THBS2,* and *INHBA* signature maybe its predictive ability in liquid biopsies since the signature genes are secreted through extracellular vesicles [32]. Further studies are needed to assess and compare the predictive potential of all these signatures in different biopsy specimens.

The delineation of *ITGA4* as a poor prognostic factor was not surprising since increased expression of integrin α_4_β_1_ is associated with tumor progression and ECM components secreted by CAFs bind integrins to activate pro-tumorigenic pathways [41, 79, 80]. However, the poor prognostic effect of *EMILIN11* and *TSPAN9* was surprising since EMILIN-1 is regarded as a tumor suppressor which acts synergistically with TSPAN9. Knockout of *EMILIN11* or suppression of EMILIN-1 - integrin α_4_β_1_ interaction was associated with decreased expression of the tumor suppressor PTEN, and increased activity of PI3K/Akt and ERK1/2 pathways, leading to hyperproliferation of dermal fibroblasts and keratinocytes, increased skin carcinogenesis and lymph node metastasis [50, 53]. Knocking out *EMILIN1* or transgenic expression of an *EMILIN1* mutant with impaired binding to integrin α_4_β_1_ increased the susceptibility of mice to develop colon cancer [81]. Based on these findings *EMILIN1* was proposed as a tumor suppressor. However, *EMILIN1* overexpression was detected in serous ovarian carcinoma, soft tissue osteosarcoma, and low-grade glioma (LGG) which are malignant tumors with high recurrence rates [54, 55, 82]. This suggests a tissue-dependent anti-tumorigenic or pro-tumorigenic role for EMILIN-1.

Recently, EMILIN-1 was suggested to increase TSPAN9 expression in gastric cancer cell lines and form a complex with TSPAN9 to synergistically inhibit FAK/Ras/Erk pathway and suppress invasion and migration. Since overexpression of only *EMILIN1* did not induce a similar anti-tumor response in gastric cancer cell lines, it was suggested that the anti-tumor effect of EMILIN-1 may be dependent on TSPAN9 [56]. Therefore, we tested whether the addition of *TSPAN9* to the *CO1A1, COL5A1, ITGA4*, and *EMILIN1* Cox model can decrease the poor prognostic impact of CAF infiltration in gastric cancer. Unexpectedly, the hazard ratio and risk score for the CAF infiltration increased further in stomach adenocarcinoma but remained zero in adrenocortical carcinoma, kidney renal papillary cell carcinoma, and mesothelioma (Table 4).

Although some studies suggest an anti-cancer role for TSPAN9 [57, 58], high TSPAN9 expression was associated with resistance to 5-fluorouracil via suppressing autophagy in gastric cancer cell lines [83]. Expression of TSPAN9 was significantly lower in gastric cancer tissue compared to adjacent normal gastric tissue in a cohort of 105 gastric cancer samples. However, the same cohort reported high TSPAN9 expression as a poor prognostic factor for survival [84]. Therefore, whether EMILIN1 and TSPAN9 exert an anti-tumor effect or pro-tumorigenic effect in gastric cancer is not clear yet.

Our investigation of TCGA stomach adenocarcinoma data does not suggest either a poor or a good prognostic role for *EMILIN1* or *TSPAN9* per se (Additional file 2: Fig. S8B-C). Despite that, the *EMILIN1* and *TSPAN9* displayed a stage-dependent increase in expression (Fig. 8f), and their hazard ratios increased by stage in parallel to the stage-dependent increase in the hazard ratio of CAFs (Additional file 2: Fig. S8E-F). Moreover, the stepwise addition of these two genes as covariates to the *CO1A1, COL5A1*, and *ITGA4* multivariate Cox model tremendously increased the hazard ratio for the poor prognostic impact of CAF infiltration, which suggested a stage and CAF dependent role for *EMILIN1* and *TSPAN9* in gastric cancer (Table 4). This may explain why these two genes display anti-tumor effects in monoculture gastric cancer cell lines but display poor prognostic effects in the gastric cancer patient cohort by Feng et al. [84] and TCGA stomach adenocarcinoma cohort we analyzed in this study.

It may be speculated that the ECM remodeling enzymes secreted from CAFs may cleave EMILIN-1 and prevent its anti-tumor action together with TSPAN9 despite their high expression in the tumor tissue. Accordingly, proteolytic cleavage of EMILIN-1 by MMPs or neutrophil elastase was suggested as a mechanism for pro-tumorigenic effect in some tumors with high *EMILIN1* expression [48, 85, 86]. For ECM proteins, it is also not unusual to serve different functions in cleaved forms vs. multimeric forms [87]. A similar mechanism may explain the context-dependent role of EMILIN-1 and TSPAN9 in gastric cancer. Although there is no evidence in the literature yet for multimerization of EMILIN-1, this may also be a possible mechanism for its context-dependent action, since our analysis pointed out “protein-crosslinking” as an enriched biological process in gastric cancer. Elucidation of these molecular and cellular mechanisms may present EMILIN-1 and TSPAN9 as new therapeutic targets in gastric cancer. This will be addressed in our future studies.

To build the poor prognostic gene signature for CAF infiltration, we analyzed the TCGA stomach adenocarcinoma data in TIMER 2.0. TIMER 2.0 implements four different algorithms, namely EPIC, MCP-Counter (Microenvironment Cell Populations-counter), Xcell, and TIDE (Tumor Immune Dysfunction and Exclusion) to predict the relative proportions of different cell populations in tumor samples [88–91]. All these algorithms device reference gene expression profiles for each cell type, established from the RNA-seq profiles of circulating immune cells and non-cancerous cells that infiltrate the tumors. One limitation to the study may be that only the TIDE algorithm predicted a poor prognostic impact for CAF infiltration in *COL1A1, COL5A1, ITGA4*, *EMILIN1*, and *TSPAN9* multivariate Cox model in gastric cancer. One reason may be that the reference gene expression profiles for CAFs in all four algorithms are different, which leads to differences in the allocation of samples to high vs. low CAF infiltration groups. Dissecting these differences in detail may improve the power of these algorithms to predict the abundance of CAFs in tumor samples. Moreover, mostly melanoma samples are used to establish reference gene expression profiles for tumor-infiltrating cells. These reference gene expression profiles may not exactly reflect the gene expression pattern for CAFs in gastric cancer or other cancers. Besides intertumoral heterogeneity, intra-tumoral heterogeneity in CAFs further complicates the picture [7]. Building tumor and subclone-specific reference gene expression profiles for CAFs may better illuminate the prognostic role of CAFs and their interactor genes in cancer. Such an approach may also reveal new molecular targets to prevent CAF infiltration in cancer.

Among the signature genes we identified in this study, targeting the COL1A1, COL5A1 and ITGA4 may have a therapeutic potential in gastric tumors with high CAF infiltration. Our search in drug databases brings out collagenase *clostridium histolyticum*, halofuginone, and ocriplasmin as agents that act on COL1A1 and COL5A1. Their anti-cancer action should be validated first in gastric cancer cell models and in vivo studies. Among these three, halofuginone seems to be the closest candidate for use as an anti-cancer agent in gastric cancer since it showed promising anti-cancer effects in other cancers. It may also prevent the formation of CAFs [68, 69]. Moreover, collagenase *clostridium histolyticum* and ocriplasmin carry risks, since cleavage of collagens may lead to a release of several growth factors that induce tumor progression. Additionally, their systemic use can be problematic in cancer due to the risk of organ and vascular toxicity [63]. Active targeting of the gastric tumors via nanocarriers or local administration may allow their use in gastric cancer. But still, there may be destructive effects on healthy gastric tissue limiting the doses that could be used in patients safely. All these points should be addressed in future studies.

Integrin α_4_β_1_ also has potential as a therapeutic target in cancer since it has a key role in metastasis, chemoresistance, angiogenesis, and lymphangiogenesis [41]. Despite that, agents targeting integrin α_4_β_1_ have not entered the clinical trials for cancer. The concerns about the risk of cancer development with targeting integrin α_4_β_1_ may be the reason since this action inhibits the migration of lymphocytes. However, the comparative analysis did not reveal an increased risk of cancer with natalizumab [92]. Uncovering the signaling mechanisms by which integrin α_4_β_1_ contributes to cancer may lead to the development of new strategies for targeting integrin α_4_β_1_ without raising the concerns about cancer development.

## Conclusion

In this study, we identified the six key CAF markers namely *COL1A1, COL1A2, COL3A1, COL5A1, FN1*, and *SPARC* in gastric cancer that can be used as predictors of CAF infiltration and prognostic biomarkers in gastric cancer. With further analysis, we revealed *COL1A1*, *COL5A1*, *ITGA4, EMILIN1,* and *TSPAN9* as a poor prognostic gene signature for CAF infiltration, with high specificity to stomach adenocarcinoma. This signature could be translated to the clinic with further studies as a predictive tool for poor prognosis. Testing the candidate drugs, we identified in this study, with further *in vitro* and *in vivo* studies may present them as potential drugs for the treatment of gastric cancer. More importantly, investigating the mechanisms by which the signature genes strengthen the poor prognostic impact of CAFs may put forth new molecular targets for the effective treatment of gastric cancer. These points will be addressed in our future studies.

## Supplementary Information

**Additional file 1: Table S1**. Characteristics of the GEO datasets used in the study. **Table S2**. Molecular functions of the differentially expressed genes in gastric cancer. **Table S3**. Functional enrichment analysis of the upregulated genes. **Table S4**. Functional enrichment analysis of the downregulated genes. **Table S5**. Topological parameters for the connected nodes in the protein-protein interaction network. **Table S6**. The KEGG pathways enriched at each module. **Table S7**. Parameters of the multivariate Cox proportional regression model for CAF with CAF marker genes. **Table S8**. Parameters of the multivariate Cox proportional regression model for CAF with two CAF gene signatures. **Table S9**. Parameters of the multivariate Cox proportional regression model for CAF with integrin α_4_β_1_ subunits. **Table S10**. Parameters of the multivariate Cox Proportional regression model for CAF with *ITGA4* partners as covariates.

**Additional file 2: Figure S1**. The protein-protein interaction network of the upregulated genes in gastric cancer. Disconnected nodes are hidden in the network. **Figure S2**. Correlation of the poor prognostic genes with cancer-associated fibroblast infiltration in gastric cancer. Correlation of the *THBS1, THBS2, INHBA, CXCL12, TGFB, VEGFB, COL10A1, AREG,* or *EFNA5* expression with the cancer-associated fibroblast infiltration in stomach adenocarcinoma (STAD). TIDE algorithm was used to analyze TCGA STAD data in TIMER2.0. **Figure S3**. KM-Survival Curve for *COL1A2* in stomach adenocarcinoma. Analysis was performed on UALCAN using TCGA data. **Figure S4**. The differential expression of six CAF markers in diffuse vs. intestinal subtypes of gastric cancer. The differential expression of A *COL1A1*, B *COL1A2*, C *COL3A1*, D *COL5A1*, E *FN1*, and F *SPARC* in diffuse vs. intestinal subtypes of gastric cancer and normal gastric tissues from corresponding patients (Abbreviated as “Normal tissue-Dif” for patients with diffuse gastric cancer and “Normal tissue-Int” for patients with intestinal gastric cancer) in the Asian Cancer Research Group gastric cancer dataset (GSE66229). Analysis was performed on GEO2R. **Figure S5**. The hazard ratio for CAF infiltration with respect to tumor stage in stomach adenocarcinoma. Bars indicate a 95% confidence interval for hazard ratios. TIDE algorithm was used to allocate TCGA stomach adenocarcinoma samples to high vs. low CAF infiltration groups in TIMER2.0. (* *p* < 0.05, *** *p* < 0.001). **Figure S6**. The interacting partners of ITGA4. Network representation for interacting partners of ITGA4 with respect to A protein types and B biological processes involved. To visualize the interacting partners of ITGA4, inBio Discover™ by Intomics A/S was used (https://inbio-discover.com/) (Intomics A/S has not endorsed the results of the published article). **Figure S7**. The differential expression of cancer-associated fibroblast poor prognostic signature genes in other cancers. Differential expression of A-C *COL1A1*, D-F *COL5A1*, G-I *ITGA4,* K-M *EMILIN1,* and N-P *TSPAN9* with respect to tumor stage in adrenocortical carcinoma (ACC), kidney renal papillary cell carcinoma (KIRP), and mesothelioma (MESO). TCGA data was analyzed on UALCAN (unpaired t-test, * *p* < 0.05, ** *p* < 0.01, *** *p* < 0.001). **Figure S8**. The prognostic impact of *ITGA4, EMILIN1,* and *TSPAN9* in gastric cancer. Kaplan-Meier survival curves for A *ITGA4,* B *EMILIN1,* and C *TSPAN9* in gastric cancer. The increase in the hazard ratio in the Cox proportional regression model for D *ITGA4,* E *EMILIN1,* and F *TSPAN9* by stage in gastric cancer. Bars indicate the 95% confidence interval for hazard ratios. TCGA stomach adenocarcinoma samples were analyzed in TIMER2.0. (* *p* < 0.05, ** *p* < 0.01, *** *p* < 0.001).

## Data Availability

The datasets we analyzed during the current study are available in the Gene Expression Omnibus (GEO) repository with the accession numbers GSE13911, GSE29272, GSE79973, and GSE118916. These datasets can be freely and openly accessed respectively at https://www.ncbi.nlm.nih.gov/geo/query/acc.cgi?acc=GSE13911, https://www.ncbi.nlm.nih.gov/geo/query/acc.cgi?acc=GSE29272, https://www.ncbi.nlm.nih.gov/geo/query/acc.cgi?acc=GSE79973, https://www.ncbi.nlm.nih.gov/geo/query/acc.cgi?acc=GSE118916 [93–96]. The studies that these datasets originated from were cited in the methods section as references 7–10.

## Abbreviations

ACC: Adrenocortical carcinoma
ACRG: Asian Cancer Research Group
Akt: Protein kinase B
AREG: Amphiregulin
ASPN: Asporin
Bcl-2: B-cell lymphoma 2
BGN: Biglycan
BIC: Bioinfo Intelligent Cloud
BLCA: Bladder urothelial carcinoma
BP: Biological process (gene ontology)
CAF: Cancer-associated fibroblast
CC: Cellular compartment (gene ontology)
COAD: Colon adenocarcinoma
COL1A1: Collagen type I alpha 1 chain
COL1A2: Collagen type I alpha 2 chain
COL2A1: Collagen type II alpha 1 chain
COL3A1: Collagen type III alpha 1 chain
COL5A1: Collagen type V alpha 1 chain
COL10A1: Collagen type X alpha 1 chain
COL11A1: Collagen type XI alpha1 chain
CXCL12: C-X-C motif chemokine ligand 12
CYP2C18: Cytochrome P450 family 2 subfamily C member 18
CYP2C9: Cytochrome P450 family 2 subfamily C member 9
CYP3A5: Cytochrome P450 Family 3 subfamily A member 5
DAVID: The Database for Annotation, Visualization, and Integrated Discovery
DEG: Differentially expressed genes
DGIdb: Drug-Gene Interaction database
ECM: Extracellular matrix
EFNA5: Ephrin A5
EMILIN1: Elastin microfibril interface-located protein 1
EMT: Epithelial-mesenchymal transition
ERK: Extracellular signal-regulated kinase
FAK: Focal adhesion kinase
FAP: Fibroblast activation protein alpha
FN1: Fibronectin 1
GBM: Glioblastoma
GC: Gastric cancer
GEO: Gene Expression Omnibus
GEPIA2: Gene Expression Profiling Interactive Analysis 2
GO: Gene Ontology
GLOBOCAN: The Global Cancer Observatory
GOTERM BP: GO-biological process
GOTERM CC: GO-cellular compartment
GOTERM MF: GO-molecular function
GOTERM: Gene Ontology Term
HER2: Human epidermal growth factor receptor 2
HNSC: Head and neck squamous carcinoma
HR: Hazard ratio
INHBA: Inhibin A
ITGA4: Integrin α4
ITGB1: Integrin β1
JAM2: Junctional adhesion molecule 2
JAM3: Junctional adhesion molecule 3
KEGG: Kyoto Encyclopedia of Genes and Genomes
KIRC: Kidney renal cell carcinoma
KIRP: Kidney renal papillary cell carcinoma
KM: Kaplan-Meier
KM plotter: Kaplan-Meier plotter
LGG: Low-grade glioma
Log FC: Log2-fold change
LOX: Lysyl oxidase
LOXL2: Lysyl oxidase-like 2
MADCAM1: Mucosal vascular addressin cell adhesion molecule 1
MAPK: Mitogen-activated protein kinase
MCODE: Molecular Complex Detection
MCP-Counter: Microenvironment Cell Populations-counter
MESO: Mesothelioma
MF: Molecular function (gene ontology)
MMP: Matrix metalloproteinase
MMP11: Matrix metalloproteinase 11
MT: Metallothionein
P53: Tumor protein p53
PD-1: Programmed cell death protein 1
PI3K: Phosphoinositide 3-kinase
PPI: Protein-protein interaction
PTEN: Phosphatase and tensin homolog
Ras: Rat sarcoma virus
RNA-seq: RNA sequencing
S100A4: S100 calcium-binding protein A4
SERPINH1: Serpin family H member 1
Smad3: SMAD Family Member 3
SPARC: Cysteine-rich acidic matrix-associated protein
SPP1: Osteopontin (secreted phosphoprotein 1)
STAD: Stomach adenocarcinoma
STRING: The Search Tool for the Retrieval of Interacting Genes/Proteins
SULF1: Sulfatase 1
TCGA: The Cancer Genome Atlas
TGFB: Transforming growth factor-beta
TGFB2: Transforming growth factor-beta 2
THBS: Thrombospondin
THBS1: Thrombospondin 1
THBS2: Thrombospondin 2
THCA: Thyroid carcinoma
TIDE: Tumor Immune Dysfunction and Exclusion
TIMER2.0: Tumor Immune Estimation Resource 2.0
TIMP1: Tissue inhibitor matrix metalloproteinase 1
TME: Tumor microenvironment
TSPAN9: Tetraspanin 9
UALCAN: University of Alabama Cancer Database
VCAM-1: Vascular cell adhesion protein 1
VCAN: Versican
VEGFB: Vascular endothelial growth factor B
VEGFR2: Vascular endothelial growth factor receptor 2
VLA-4: Very late antigen-4
