# Supplementary material for: Comprehensive bioinformatic analysis reveals a cancer-associated fibroblast gene signature as a poor prognostic factor and potential therapeutic target in gastric cancer
Source: BMC Cancer. 2022 Jun 23;22:692. doi: 10.1186/s12885-022-09736-5 (PMC9229147; doi:10.1186/s12885-022-09736-5)
Supplement: Supplementary file 1 — Additional file 1: Table S1. Characteristics of the GEO datasets used in the study. Table S2. Molecular functions of the differentially expressed genes in gastric cancer. Table S3. Functional enrichment analysis of the upregulated genes. Table S4. Functional enrichment analysis of the downregulated genes. Table S5. Topological parameters for the connected nodes in the protein-protein interaction network. Table S6. The KEGG pathways enriched at each module. Table S7. Parameters of the multivariate Cox proportional regression model for CAF with CAF marker genes. Table S8. Parameters of the multivariate Cox proportional regression model for CAF with two CAF gene signatures. Table S9. Parameters of the multivariate Cox proportional regression model for CAF with integrin α4β1 subunits. Table S10. Parameters of the multivariate Cox Proportional regression model for CAF with ITGA4 partners as covariates. [file 12885_2022_9736_MOESM1_ESM.docx]

**ADDITIONAL FILE 1:**

**SUPPLEMENTARY TABLES**

**Table S1.** Characteristics of the GEO datasets used in the study.

| **Dataset** | **Platform** | **Source** | **Normal Tissue** | **Cancer Tissue** | **Neoadjuvant Chemo** |
| --- | --- | --- | --- | --- | --- |
| GSE13911 | Affymetrix Human Genome U133 Plus 2.0 Array | Stomach | 31 | 38 | No |
| GSE29272 | Affymetrix Human Genome U133A Array | Stomach | 134 | 134 | No |
| GSE79973 | Affymetrix Human Genome U133 Plus 2.0 Array | Stomach | 10 | 10 | No |
| GSE118916 | Affymetrix Human Gene Expression Array | Stomach | 25 | 25 | No |

**Table S2.** Molecular functions of the differentially expressed genes in gastric cancer.

| **UPREGULATED GENES** | |
| --- | --- |
| ***Gene Symbol*** | **Molecular Function** |
| ***RAB31*** | GTPase activity, GTP binding, GDP binding |
| ***TIMP1*** | protease binding, cytokine activity, protein binding, growth factor activity, metalloendopeptidase inhibitor activity, metal ion binding |
| ***THY1*** | GTPase activator activity, integrin binding, protein binding, protein kinase binding, GPI anchor binding |
| ***APOC1*** | phospholipase inhibitor activity, fatty acid-binding, phosphatidylcholine binding, lipase inhibitor activity, phosphatidylcholine-sterol O-acyltransferase activator activity |
| ***ASPN*** | protein kinase inhibitor activity, calcium ion binding, collagen-binding |
| ***BGN*** | protein kinase inhibitor activity, extracellular matrix structural constituent, glycosaminoglycan binding, extracellular matrix binding |
| ***CDH11*** | calcium ion binding |
| ***COL1A1*** | extracellular matrix structural constituent, protein binding, identical protein binding, metal ion binding, platelet-derived growth factor binding |
| ***COL1A2*** | extracellular matrix structural constituent, protein binding, protein binding, bridging, identical protein binding, SMAD binding, metal ion binding, platelet-derived growth factor binding |
| ***COL3A1*** | integrin binding, extracellular matrix structural constituent, protein binding, SMAD binding, metal ion binding, platelet-derived growth factor binding |
| ***COL4A1*** | extracellular matrix structural constituent, protein binding, extracellular matrix constituent conferring elasticity, platelet-derived growth factor binding |
| ***COL5A1*** | integrin binding, extracellular matrix structural constituent, heparin-binding, proteoglycan binding, metal ion binding, platelet-derived growth factor binding |
| ***COL5A2*** | extracellular matrix structural constituent, SMAD binding, metal ion binding |
| ***COL6A3*** | serine-type endopeptidase inhibitor activity |
| ***COL10A1*** | protein binding, metal ion binding |
| ***ECT2*** | signal transducer activity, guanyl-nucleotide exchange factor activity, Rho guanyl-nucleotide exchange factor activity, GTPase activator activity, protein binding, Rho GTPase binding, protein homodimerization activity |
| ***FBN1*** | integrin binding, hormone activity, extracellular matrix structural constituent, calcium ion binding, protein binding, heparin-binding, extracellular matrix constituent conferring elasticity, protein complex binding |
| ***FAP*** | protease binding, endopeptidase activity, metalloendopeptidase activity, serine-type endopeptidase activity, integrin binding, protein binding, peptidase activity, serine-type peptidase activity, dipeptidyl-peptidase activity, protein homodimerization activity, protein dimerization activity |
| ***FN1*** | protease binding, integrin binding, protein binding, collagen binding, heparin-binding, peptidase activator activity, identical protein binding |
| ***LGALS1*** | glycoprotein binding, signal transducer activity, protein binding, lactose binding, protein homodimerization activity, laminin-binding, poly(A) RNA binding |
| ***INHBA*** | cytokine activity, transforming growth factor-beta receptor binding, hormone activity, protein binding, growth factor activity, peptide hormone binding, inhibin binding, identical protein binding, protein heterodimerization activity, type II activin receptor binding, binding, protein heterodimerization activity, type II activin receptor binding |
| ***IGF2BP3*** | Nucleotide-binding, nucleic acid binding, RNA binding, mRNA 3'-UTR binding, protein binding, poly(A) RNA binding, translation regulator activity, mRNA 5'-UTR binding |
| ***IGFBP7*** | protein binding, insulin-like growth factor binding |
| ***MEST*** | catalytic activity, hydrolase activity |
| ***MFAP2*** | component of elastin microfibrils in the extracellular matrix |
| ***NNMT*** | nicotinamide N-methyltransferase activity, pyridine N-methyltransferase activity |
| ***NID2*** | calcium ion binding, protein binding, collagen-binding |
| ***OLFML2B*** | extracellular matrix binding |
| ***PMEPA1*** | protein binding, WW domain binding, R-SMAD binding |
| ***RARRES1*** | Inhibitor of the cytoplasmic carboxypeptidase AGBL2 may regulate the alpha-tubulin tyrosination cycle |
| ***SFRP4*** | G-protein coupled receptor activity, protein binding, Wnt-protein binding, Wnt-activated receptor activity |
| ***SPP1*** | cytokine activity, protein binding, extracellular matrix binding |
| ***SPARC*** | calcium ion binding, protein binding, collagen binding, extracellular matrix binding |
| ***SERPINH1*** | serine-type endopeptidase inhibitor activity, collagen binding, poly(A) RNA binding, unfolded protein binding |
| ***SULF1*** | catalytic activity, arylsulfatase activity, calcium ion binding, N-acetylglucosamine-6-sulfatase activity, sulfuric ester hydrolase activity |
| ***THBS1*** | phosphatidylserine binding, glycoprotein binding, fibronectin-binding, integrin binding, calcium ion binding, protein binding, heparin-binding, fibroblast growth factor binding, low-density lipoprotein particle binding, identical protein binding, laminin-binding, proteoglycan binding, transforming growth factor-beta binding, extracellular matrix binding, fibrinogen binding, collagen V binding |
| ***THBS2*** | calcium ion binding, protein binding, heparin-binding |
| ***VCAN*** | extracellular matrix structural constituent, calcium ion binding, protein binding, glycosaminoglycan binding, hyaluronic acid-binding, carbohydrate-binding |
| **DOWNREGULATED GENES** | |
| ***Gene Symbol*** | **Molecular Function** |
| ***HMGCS2*** | hydroxymethylglutaryl-CoA synthase activity |
| ***ATP4A*** | Nucleotide-binding, magnesium ion binding, sodium:potassium-exchanging ATPase activity, ATP binding, hydrogen:potassium-exchanging ATPase activity, metal ion binding |
| ***ATP4B*** | protein binding, hydrogen:potassium-exchanging ATPase activity |
| ***GPRC5C*** | G-protein coupled receptor activity |
| ***HRASLS2*** | transferase activity, transferring acyl groups, hydrolase activity |
| ***NQO1*** | NAD(P)H dehydrogenase (quinone) activity, cytochrome-b5 reductase activity, acting on NAD(P)H, superoxide dismutase activity, protein binding, identical protein binding, poly(A) RNA binding |
| ***ALDH3A1*** | 3-chloroallyl aldehyde dehydrogenase activity, aldehyde dehydrogenase (NAD) activity, aldehyde dehydrogenase [NAD(P)+] activity, protein binding, alcohol dehydrogenase (NADP+) activity, oxidoreductase activity, oxidoreductase activity, acting on the aldehyde or oxo group of donors, NAD or NADP as acceptor, benzaldehyde dehydrogenase (NAD+) activity |
| ***AKR1B10*** | retinal dehydrogenase activity, aldo-keto reductase (NADP) activity, ~protein binding, geranylgeranyl reductase activity, indanol dehydrogenase activity |
| ***AKR1C1*** | alditol: NADP+ 1-oxidoreductase activity, aldo-keto reductase (NADP) activity, protein binding, oxidoreductase activity, oxidoreductase activity, acting on NAD(P)H, quinone or similar compound as acceptor, phenanthrene 9,10-monooxygenase activity, carboxylic acid-binding, bile acid-binding, 17-alpha,20-alpha-dihydroxypregn-4-en-3-one dehydrogenase activity, androsterone dehydrogenase (B-specific) activity, ketosteroid monooxygenase activity, trans-1,2-dihydrobenzene-1,2-diol dehydrogenase activity, indanol dehydrogenase activity |
| ***AKR1C2*** | alditol: NADP+ 1-oxidoreductase activity, oxidoreductase activity, oxidoreductase activity, acting on NAD(P)H, quinone or similar compound as acceptor, phenanthrene 9,10-monooxygenase activity, carboxylic acid-binding, bile acid-binding, androsterone dehydrogenase (A-specific) activity, ketosteroid monooxygenase activity, trans-1,2-dihydrobenzene-1,2-diol dehydrogenase activity |
| ***ALDOB*** | fructose-bisphosphate aldolase activity, protein binding, cytoskeletal protein binding, identical protein binding, ATPase binding, fructose-1-phosphate aldolase activity, fructose binding |
| ***AZGP1*** | glycoprotein binding, antigen binding, ribonuclease activity, protein binding, protein transmembrane transporter activity |
| ***CAPN9*** | calcium-dependent cysteine-type endopeptidase activity, calcium ion binding |
| ***CPA2*** | carboxypeptidase activity, metallocarboxypeptidase activity, zinc ion binding |
| ***CCKBR*** | phosphatidylinositol phospholipase C activity, cholecystokinin receptor activity, protein binding, gastrin receptor activity, type B gastrin/cholecystokinin receptor binding, 1-phosphatidylinositol-3-kinase regulator activity |
| ***COL2A1*** | extracellular matrix structural constituent, extracellular matrix structural constituent conferring tensile strength, identical protein binding, metal ion binding, platelet-derived growth factor binding |
| ***CKMT2*** | creatine kinase activity, ATP binding, kinase activity |
| ***CYP2C18*** | monooxygenase activity, iron ion binding, arachidonic acid epoxygenase activity, steroid hydroxylase activity, oxidoreductase activity, acting on paired donors, with incorporation or reduction of molecular oxygen, oxygen binding, heme binding, aromatase activity |
| ***CYP2C9*** | cholesterol 25-hydroxylase activity, monooxygenase activity, iron ion binding, drug binding, arachidonic acid epoxygenase activity, steroid hydroxylase activity, oxidoreductase activity, oxidoreductase activity, acting on paired donors, with incorporation or reduction of molecular oxygen, (S)-limonene 6-monooxygenase activity, (S)-limonene 7-monooxygenase activity, oxygen binding, heme binding, caffeine oxidase activity, (R)-limonene 6-monooxygenase activity |
| ***CYP3A5*** | monooxygenase activity, iron ion binding, oxidoreductase activity, oxidoreductase activity, acting on paired donors, with incorporation or reduction of molecular oxygen, oxidoreductase activity, acting on paired donors, with incorporation or reduction of molecular oxygen, reduced flavin or flavoprotein as one donor, and incorporation of one atom of oxygen, oxygen binding, heme binding, aromatase activity |
| ***DGKD*** | diacylglycerol kinase activity, protein binding, ATP binding, kinase activity, diacylglycerol binding, protein homodimerization activity, metal ion binding, protein heterodimerization activity |
| ***EPB41L4B*** | structural constituent of the cytoskeleton, cytoskeletal protein binding |
| ***ESRRG*** | RNA polymerase II regulatory region sequence-specific DNA binding, transcriptional activator activity, RNA polymerase II transcription regulatory region sequence-specific binding, DNA binding, transcription factor activity, sequence-specific DNA binding, steroid hormone receptor activity, retinoic acid receptor activity, steroid binding, protein binding, zinc ion binding, sequence-specific DNA binding, AF-2 domain binding |
| ***ETNPPL*** | transaminase activity, pyridoxal phosphate binding, identical protein binding, ethanolamine-phosphate phospho-lyase activity |
| ***FOLR1*** | receptor activity, folic acid-binding, drug binding, folic acid transporter activity, methotrexate binding, folic acid receptor activity |
| ***GIF*** | cobalamin binding |
| ***GKN1*** | mitogenic activity and may be involved in maintaining the integrity of the gastric mucosal epithelium |
| ***HDC*** | histidine decarboxylase activity, protein binding, carboxy-lyase activity, pyridoxal phosphate binding |
| ***HPGD*** | catalytic activity, prostaglandin E receptor activity, 15-hydroxyprostaglandin dehydrogenase (NAD+) activity, oxidoreductase activity, protein homodimerization activity, NAD binding, NAD+ binding |
| ***MAL*** | protein binding, lipid binding, channel activity, peptidase activator activity involved in the apoptotic process, structural constituent of the myelin sheath |
| ***MT1E*** | zinc ion binding, metal ion binding |
| ***MT1F*** | protein binding, zinc ion binding, metal ion binding |
| ***MT1G*** | protein binding, zinc ion binding, metal ion binding |
| ***MT1H*** | protein binding, zinc ion binding, metal ion binding |
| ***MT1M*** | zinc ion binding, metal ion binding |
| ***MT1X*** | protein binding, zinc ion binding, cadmium ion binding, metal ion binding |
| ***MYRF*** | DNA binding, transcription factor activity, sequence-specific DNA binding, peptidase activity |
| ***NEDD4L*** | ubiquitin-protein transferase activity, protein binding, potassium channel regulator activity, ligase activity, sodium channel regulator activity, potassium channel inhibitor activity, sodium channel inhibitor activity, ion channel binding |
| ***PXMP2*** | protein binding |
| ***PBLD*** | catalytic activity, protein binding, isomerase activity, identical protein binding |
| ***PLLP*** | protein binding, structural constituent of the myelin sheath |
| ***KCNJ15*** | inward rectifier potassium channel activity, protein binding, G-protein activated inward rectifier potassium channel activity |
| ***KCNJ16*** | inward rectifier potassium channel activity, G-protein activated inward rectifier potassium channel activity |
| ***TMPRSS2*** | serine-type endopeptidase activity, scavenger receptor activity, protein binding, serine-type peptidase activity |
| ***UBL3*** | ubiquitin-like protein 3, Drosophila homolog, highly conserved in man and C Elegans |

**Table S3.** Functional enrichment analysis of the upregulated genes.

| **Category** | **Term** | **Count** | **P-value** |
| --- | --- | --- | --- |
|  |  |  |  |
| BP | extracellular matrix organization | 18 | 8.78E-24 |
| CC | extracellular matrix | 17 | 1.45E-19 |
| CC | extracellular region | 24 | 8.52E-16 |
| CC | proteinaceous extracellular matrix | 14 | 2.97E-15 |
| MF | extracellular matrix structural constituent | 9 | 8.64E-13 |
| CC | collagen trimer | 9 | 1.06E-11 |
| BP | collagen catabolic process | 8 | 7.84E-11 |
| CC | endoplasmic reticulum lumen | 10 | 1.28E-10 |
| BP | cell adhesion | 13 | 1.50E-10 |
| CC | extracellular space | 17 | 2.11E-09 |
| MF | platelet-derived growth factor binding | 5 | 5.06E-09 |
| CC | basement membrane | 7 | 1.14E-08 |
| BP | skeletal system development | 8 | 1.73E-08 |
| BP | collagen fibril organization | 6 | 2.14E-08 |
| MF | integrin binding | 7 | 7.03E-08 |
| MF | extracellular matrix binding | 5 | 2.24E-07 |
| BP | cellular response to amino acid stimulus | 5 | 3.32E-06 |
| MF | collagen binding | 5 | 6.95E-06 |
| CC | extracellular exosome | 18 | 1.07E-05 |
| BP | collagen biosynthetic process | 3 | 7.05E-05 |
| MF | calcium ion binding | 9 | 8.64E-05 |
| CC | platelet alpha granule lumen | 4 | 1.88E-04 |
| MF | Heparin-binding | 5 | 3.24E-04 |
| BP | negative regulation of angiogenesis | 4 | 3.41E-04 |
| CC | platelet alpha granule | 3 | 3.59E-04 |
| BP | extracellular matrix disassembly | 4 | 6.20E-04 |
| CC | cell surface | 7 | 7.14E-04 |
| BP | wound healing | 4 | 7.20E-04 |
| BP | ossification | 4 | 7.20E-04 |
| BP | platelet degranulation | 4 | 0.001497 |
| BP | negative regulation of endothelial cell proliferation | 3 | 0.001847 |
| BP | skin development | 3 | 0.002535 |
| BP | blood vessel development | 3 | 0.003159 |
| MF | SMAD binding | 3 | 0.003575 |
| CC | collagen type I trimer | 2 | 0.004057 |
| BP | response to peptide hormone | 3 | 0.004216 |
| BP | response to drug | 5 | 0.004345 |
| BP | negative regulation of endodermal cell differentiation | 2 | 0.004402 |
| BP | peptide cross-linking | 3 | 0.005415 |
| BP | response to cytokine | 3 | 0.005845 |
| CC | collagen type V trimer | 2 | 0.006079 |
| BP | eye morphogenesis | 2 | 0.006596 |
| BP | cartilage development | 3 | 0.007469 |
| BP | post-embryonic eye morphogenesis | 2 | 0.008785 |
| MF | extracellular matrix constituent conferring elasticity | 2 | 0.010325 |
| BP | basement membrane organization | 2 | 0.017496 |
| CC | fibrinogen complex | 2 | 0.018129 |
| MF | protease binding | 3 | 0.018549 |
| BP | skin morphogenesis | 2 | 0.019662 |
| BP | negative regulation of protein kinase activity | 3 | 0.020042 |
| CC | microfibril | 2 | 0.020123 |
| BP | embryonic eye morphogenesis | 2 | 0.021823 |
| BP | negative regulation of fibroblast growth factor receptor signaling pathway | 2 | 0.021823 |
| BP | osteoblast differentiation | 3 | 0.021977 |
| MF | proteoglycan binding | 2 | 0.022578 |
| CC | endoplasmic reticulum | 6 | 0.024995 |
| BP | extracellular fibril organization | 2 | 0.026132 |
| BP | dermatan sulfate biosynthetic process | 2 | 0.026132 |
| BP | platelet activation | 3 | 0.026495 |
| BP | negative regulation of endopeptidase activity | 3 | 0.029104 |
| BP | leukocyte migration | 3 | 0.029549 |
| BP | chondroitin sulfate catabolic process | 2 | 0.030422 |
| BP | negative regulation of cell-substrate adhesion | 2 | 0.030422 |
| BP | protein heterotrimerization | 2 | 0.030422 |
| MF | glycosaminoglycan binding | 2 | 0.036688 |
| MF | Laminin-binding | 2 | 0.050599 |
| MF | cytokine activity | 3 | 0.051379 |
| BP | chondroitin sulfate biosynthetic process | 2 | 0.053691 |
| BP | endochondral ossification | 2 | 0.055779 |
| BP | negative regulation of cell proliferation | 4 | 0.05606 |
| BP | endodermal cell differentiation | 2 | 0.057863 |
| BP | odontogenesis | 2 | 0.057863 |
| BP | regulation of immune response | 3 | 0.05843 |
| BP | heart development | 3 | 0.061351 |
| BP | glycosaminoglycan metabolic process | 2 | 0.062017 |
| BP | embryonic skeletal system development | 2 | 0.064087 |
| CC | membrane raft | 3 | 0.065424 |
| MF | identical protein binding | 5 | 0.067884 |
| BP | negative regulation of JAK-STAT cascade | 2 | 0.084548 |
| BP | angiogenesis | 3 | 0.08639 |
| BP | bone development | 2 | 0.088587 |
| BP | response to hormone | 2 | 0.088587 |
| BP | embryo implantation | 2 | 0.088587 |
| BP | response to unfolded protein | 2 | 0.088587 |
| BP | cellular response to growth factor stimulus | 2 | 0.094614 |
| BP | positive regulation of endothelial cell migration | 2 | 0.096615 |
| BP | response to cAMP | 2 | 0.096615 |
| **Category** | **Term** | **Count** | **P-value** |
| KEGG Pathway | ECM-receptor interaction | 11 | 2.46E-15 |
| KEGG Pathway | Focal adhesion | 11 | 1.66E-11 |
| KEGG Pathway | Protein digestion and absorption | 8 | 1.25E-09 |
| KEGG Pathway | PI3K-Akt signaling pathway | 11 | 2.71E-09 |
| KEGG Pathway | Amoebiasis | 7 | 1.86E-07 |
| KEGG Pathway | Platelet activation | 5 | 3.03E-04 |

BP: GO-biological process, MF: GO-molecular function, and CC: GO-cellular compartment.

**Table S4.** Functional enrichment analysis of the downregulated genes.

| **GO Category** | **Term** | **Count** | **P-value** |
| --- | --- | --- | --- |
| BP | cellular response to zinc ion | 6 | 8.67E-10 |
| BP | negative regulation of growth | 6 | 8.67E-10 |
| BP | cellular response to cadmium ion | 5 | 7.86E-08 |
| BP | digestion | 6 | 4.83E-07 |
| BP | xenobiotic metabolic process | 6 | 1.41E-06 |
| MF | oxidoreductase activity | 6 | 1.32E-04 |
| BP | steroid metabolic process | 4 | 1.67E-04 |
| BP | doxorubicin metabolic process | 3 | 1.69E-04 |
| BP | daunorubicin metabolic process | 3 | 1.69E-04 |
| BP | oxidation-reduction process | 8 | 6.02E-04 |
| BP | potassium ion import | 3 | 0.002215 |
| MF | oxygen binding | 3 | 0.006085 |
| MF | indanol dehydrogenase activity | 2 | 0.007446 |
| MF | hydrogen:potassium-exchanging ATPase activity | 2 | 0.007446 |
| MF | ketosteroid monooxygenase activity | 2 | 0.007446 |
| MF | oxidoreductase activity, acting on paired donors, with incorporation or reduction of molecular oxygen | 3 | 0.008845 |
| MF | monooxygenase activity | 3 | 0.009146 |
| MF | phenanthrene 9,10-monooxygenase activity | 2 | 0.009916 |
| MF | trans-1,2-dihydrobenzene-1,2-diol dehydrogenase activity | 2 | 0.009916 |
| BP | cellular response to jasmonic acid stimulus | 2 | 0.009968 |
| BP | drug catabolic process | 2 | 0.014916 |
| CC | perinuclear region of cytoplasm | 6 | 0.01641 |
| MF | carboxylic acid binding | 2 | 0.01729 |
| MF | alditol:NADP+ 1-oxidoreductase activity | 2 | 0.01729 |
| BP | potassium ion transport | 3 | 0.017875 |
| CC | organelle membrane | 3 | 0.018717 |
| MF | oxidoreductase activity, acting on NAD(P)H, quinone, or similar compound as acceptor | 2 | 0.019736 |
| MF | bile acid binding | 2 | 0.019736 |
| BP | membrane raft polarization | 2 | 0.019839 |
| BP | progesterone metabolic process | 2 | 0.022292 |
| MF | zinc ion binding | 8 | 0.024002 |
| MF | structural constituent of the myelin sheath | 2 | 0.02461 |
| MF | G-protein activated inward rectifier potassium channel activity | 2 | 0.02461 |
| BP | response to metal ion | 2 | 0.024739 |
| BP | cellular aldehyde metabolic process | 2 | 0.02718 |
| BP | oxidative demethylation | 2 | 0.029614 |
| CC | extracellular exosome | 13 | 0.030865 |
| BP | regulation of ion transmembrane transport | 3 | 0.031401 |
| BP | prostaglandin metabolic process | 2 | 0.032043 |
| MF | aldo-keto reductase (NADP) activity | 2 | 0.036692 |
| MF | arachidonic acid epoxygenase activity | 2 | 0.036692 |
| BP | epoxygenase P450 pathway | 2 | 0.044099 |
| MF | heme binding | 3 | 0.04558 |
| BP | cellular sodium ion homeostasis | 2 | 0.046492 |
| MF | inward rectifier potassium channel activity | 2 | 0.048628 |
| CC | integral component of plasma membrane | 8 | 0.051297 |
| MF | iron ion binding | 3 | 0.055502 |
| MF | steroid hydroxylase activity | 2 | 0.062761 |
| MF | aromatase activity | 2 | 0.065096 |
| CC | basolateral plasma membrane | 3 | 0.070069 |
| BP | ion transmembrane transport | 3 | 0.096828 |
| **Category** | **Term** | **Count** | **P-value** |
| KEGG Pathway | Mineral absorption | 6 | 1.50E-06 |
| KEGG Pathway | Gastric acid secretion | 5 | 3.35E-04 |
| KEGG Pathway | Metabolism of xenobiotics by cytochrome P450 | 5 | 3.53E-04 |
| KEGG Pathway | Chemical carcinogenesis | 4 | 0.005893 |
| KEGG Pathway | Steroid hormone biosynthesis | 3 | 0.029477 |
| KEGG Pathway | Retinol metabolism | 3 | 0.035336 |
| KEGG Pathway | Drug metabolism - cytochrome P450 | 3 | 0.039474 |
| KEGG Pathway | Metabolic pathways | 11 | 0.044588 |
| KEGG Pathway | Histidine metabolism | 2 | 0.097635 |

BP: GO-biological process, MF: GO-molecular function, and CC: GO-cellular compartment.

**Table S5.** Topological parameters for the connected nodes in the protein-protein interaction network.

| Gene Symbol | Degree | Betweenness of Centrality | Closeness of Centrality | Clustering Coefficient | Average Shortest Path Length | Neighborhood Connectivity |
| --- | --- | --- | --- | --- | --- | --- |
| *COL3A1* | 16 | 0.25 | 0.71 | 0.52 | 1.41 | 10.12 |
| *FN1* | 16 | 0.20 | 0.69 | 0.43 | 1.44 | 9.68 |
| *COL1A2* | 15 | 0.09 | 0.68 | 0.59 | 1.48 | 10.80 |
| *COL1A1* | 15 | 0.06 | 0.68 | 0.62 | 1.48 | 11.26 |
| *COL5A1* | 13 | 0.02 | 0.64 | 0.76 | 1.56 | 12.15 |
| *SPARC* | 13 | 0.06 | 0.64 | 0.58 | 1.56 | 11.38 |
| *COL4A1* | 12 | 0.08 | 0.61 | 0.74 | 1.63 | 11.66 |
| *FBN1* | 12 | 0.11 | 0.61 | 0.45 | 1.63 | 10.33 |
| *COL5A2* | 12 | 0.02 | 0.61 | 0.76 | 1.63 | 11.58 |
| *SERPINH1* | 11 | 0.01 | 0.60 | 0.89 | 1.67 | 12.72 |
| *COL2A1* | 10 | 0.00 | 0.54 | 0.96 | 1.85 | 12.50 |
| *TIMP1* | 9 | 0.02 | 0.54 | 0.61 | 1.85 | 9.55 |
| *COL6A3* | 9 | 0.00 | 0.51 | 1.00 | 1.96 | 12.55 |
| *COL10A1* | 9 | 0.00 | 0.51 | 1.00 | 1.96 | 12.55 |
| *BGN* | 8 | 0.04 | 0.56 | 0.57 | 1.78 | 12.12 |
| *VCAN* | 7 | 0.00 | 0.47 | 0.76 | 2.11 | 9.14 |
| *SPP1* | 7 | 0.00 | 0.48 | 0.81 | 2.07 | 9.85 |
| *IGFBP7* | 6 | 0.00 | 0.47 | 1.00 | 2.15 | 9.50 |
| *LGALS1* | 6 | 0.00 | 0.47 | 1.00 | 2.15 | 9.50 |
| *MT1E* | 5 | 0.40 | 1.00 | 0.60 | 1.00 | 3.40 |
| *THBS1* | 4 | 0.01 | 0.46 | 0.50 | 2.19 | 10.00 |
| *MT1X* | 4 | 0.00 | 0.83 | 1.00 | 1.20 | 4.25 |
| *MT1H* | 4 | 0.00 | 0.83 | 1.00 | 1.20 | 4.25 |
| *CDH11* | 4 | 0.00 | 0.46 | 1.00 | 2.19 | 14.50 |
| *MT1F* | 4 | 0.00 | 0.83 | 1.00 | 1.20 | 4.25 |
| *MT1G* | 4 | 0.00 | 0.83 | 1.00 | 1.20 | 4.25 |
| *MFAP2* | 2 | 0.00 | 0.40 | 0.00 | 2.48 | 10.00 |
| *ASPN* | 2 | 0.07 | 0.44 | 0.00 | 2.30 | 8.50 |
| *THBS2* | 2 | 0.00 | 0.43 | 0.00 | 2.33 | 9.50 |
| *CYP3A5* | 2 | 1.00 | 1.00 | 0.00 | 1.00 | 1.00 |
| *CAPN9* | 1 | 0.00 | 1.00 | 0.00 | 1.00 | 1.00 |
| *NID2* | 1 | 0.00 | 0.39 | 0.00 | 2.59 | 12.00 |
| *MT1M* | 1 | 0.00 | 0.56 | 0.00 | 1.80 | 5.00 |
| *AKR1C2* | 1 | 0.00 | 1.00 | 0.00 | 1.00 | 1.00 |
| *CYP2C18* | 1 | 0.00 | 0.67 | 0.00 | 1.50 | 2.00 |
| *CYP2C9* | 1 | 0.00 | 0.67 | 0.00 | 1.50 | 2.00 |
| *GKN1* | 1 | 0.00 | 1.00 | 0.00 | 1.00 | 1.00 |
| *AKR1C1* | 1 | 0.00 | 1.00 | 0.00 | 1.00 | 1.00 |
| *ATP4A* | 1 | 0.00 | 1.00 | 0.00 | 1.00 | 1.00 |
| *SULF1* | 1 | 0.00 | 0.42 | 0.00 | 2.37 | 16.00 |
| *ATP4B* | 1 | 0.00 | 1.00 | 0.00 | 1.00 | 1.00 |
| *KCNJ16* | 1 | 0.00 | 1.00 | 0.00 | 1.00 | 1.00 |
| *FAP* | 1 | 0.00 | 0.42 | 0.00 | 2.41 | 16.00 |
| *KCNJ15* | 1 | 0.00 | 1.00 | 0.00 | 1.00 | 1.00 |
| *SFRP4* | 1 | 0.00 | 0.31 | 0.00 | 3.26 | 2.00 |

**Table S6.** The KEGG pathways enriched at each module.

| **Modules** | **Enriched KEGG Pathway** | **Count** | **P-value** | **Benjamini** |
| --- | --- | --- | --- | --- |
| **1** | ECM-receptor interaction | 8 | 3.2E-13 | 1.3E-12 |
|  | Focal adhesion | 8 | 1.5E-10 | 1.5E-10 |
|  | PI3K-Akt signaling pathway | 8 | 5.8E-9 | 9.2E-9 |
| **2** | ECM-receptor interaction | 2 | 3.7E-2 | 4.5E-1 |
|  | Focal adhesion | 2 | 8.7E-2 | 5.2E-1 |
| **3** | Mineral absorption | 5 | 1.5E-9 | 1.5E-9 |

**Table S7** Parameters of the multivariate Cox proportional regression model for CAF with CAF marker genes.

| **Variates** | **HR of CAF infiltration** | **95% CI** | **z-score** | **p-value** |
| --- | --- | --- | --- | --- |
| **CAF, *COL11A1*** | 5.395 | 1.934-15.047 | 3.221 | 0.001 |
| **CAF, *FAP*** | 5.301 | 1.264-22.226 | 2.28 | 0.023 |
| **CAF, *INHBA*** | 5.428 | 1.506-19.561 | 2.586 | 0.010 |
| **CAF, *MMP11*** | 4.776 | 1.726-13.216 | 3.011 | 0.003 |
| **CAF, *S100A4*** | 6.437 | 2.392-17.320 | 3.687 | 0.000 |
| **CAF, *THBS2*** | 5.801 | 1.312-25.643 | 2.318 | 0.020 |

TCGA data for stomach adenocarcinoma samples were analyzed in the TIMER2.0 immune association - gene outcome module using the TIDE algorithm for the allocation of samples to the high vs. low CAF infiltration groups. Likelihood ratio and Score log-rank tests were performed (CAF: cancer-associated fibroblast, CI: confidence interval, HR: hazard ratio).

**Table S8.** Parameters of the multivariate Cox proportional regression model for CAF with two CAF gene signatures.

| **Variates** | **HR of CAF infiltration** | **95% CI** | **z-score** | **p-value** |
| --- | --- | --- | --- | --- |
| CAF, *THBS1, THBS2, INHBA* | 3.534 | 0.623-20.062 | 1.425 | 0.154 |
| CAF, *TGFB2, VEGFB, COL10A1, AREG, EFNA5* | 1.689 | 0.324-8.800 | 0.622 | 0.534 |

TCGA data for stomach adenocarcinoma samples were analyzed in the TIMER2.0 immune association - gene outcome module using the TIDE algorithm for the allocation of samples to the high vs. low CAF infiltration groups. Likelihood ratio and Score log-rank tests were performed (CAF: cancer-associated fibroblast, CI: confidence interval, HR: hazard ratio).

**Table S9.** Parameters of the multivariate Cox proportional regression model for CAF with integrin α_4_β_1_ subunits.

| **Variates** | **HR of CAF infiltration** | **95% CI** | **z-score** | **p-value** |
| --- | --- | --- | --- | --- |
| CAF, ***COL1A1, COL5A1, ITGB1*** | 10.427 | 1.862-58.408 | 2.667 | 0.008 |
| CAF, ***COL1A1, COL5A1, ITGB1, ITGA4*** | 11.270 | 1.983-64.037 | 2.733 | 0.006 |

TCGA data for stomach adenocarcinoma samples were analyzed in the TIMER2.0 immune association - gene outcome module using the TIDE algorithm for the allocation of samples to the high vs. low CAF infiltration groups. Likelihood ratio and Score log-rank tests were performed (CAF: cancer-associated fibroblast, CI: confidence interval, HR: hazard ratio).

**Table S10.** Parameters of the multivariate Cox Proportional regression model for CAF with *ITGA4* partners as covariates.

| **Variates** | **HR of CAF infiltration** | **95% CI** | **z-score** | **p-value** |
| --- | --- | --- | --- | --- |
| **CAF, *COL1A1, COL5A1, ITGA4, EMILIN1*** | 28.315 | 4.143-193.524 | 3.409 | 0.001 |
| **CAF, *COL1A1, COL5A1, ITGA4, FN1*** | 10.217 | 1.719-60.744 | 2.555 | 0.011 |
| **CAF, *COL1A1, COL5A1, ITGA4, JAM2*** | 12.806 | 1.186-138.279 | 2.101 | 0.036 |
| **CAF, *COL1A1, COL5A1, ITGA4, JAM3*** | 6.812 | 0.488-95.174 | 1.426 | 0.154 |
| **CAF, *COL1A1, COL5A1, ITGA4, MADCAM1*** | 13.100 | 2.374-72.284 | 2.952 | 0.003 |
| **CAF, *COL1A1, COL5A1, ITGA4, SPP1*** | 12.322 | 2.348-64.647 | 2.969 | 0.003 |
| **CAF, *COL1A1, COL5A1, ITGA4, THBS1*** | 7.162 | 1.016-50.466 | 1.976 | 0.048 |
| **CAF, *COL1A1, COL5A1, ITGA4, VCAM1*** | 14.496 | 2.539-82.746 | 3.009 | 0.003 |

TCGA data for stomach adenocarcinoma samples were analyzed in the TIMER2.0 immune association - gene outcome module using the TIDE algorithm for the allocation of samples to the high vs. low CAF infiltration groups. Likelihood ratio and Score log-rank tests were performed (CAF: cancer-associated fibroblast, CI: confidence interval, HR: hazard ratio).
