# Supplementary material for: Comprehensive bioinformatic analysis reveals a cancer-associated fibroblast gene signature as a poor prognostic factor and potential therapeutic target in gastric cancer
Source: BMC Cancer. 2022 Jun 23;22:692. doi: 10.1186/s12885-022-09736-5 (PMC9229147; doi:10.1186/s12885-022-09736-5)
Supplement: Supplementary file 2 — Additional file 2: Figure S1. The protein-protein interaction network of the upregulated genes in gastric cancer. Disconnected nodes are hidden in the network. Figure S2. Correlation of the poor prognostic genes with cancer-associated fibroblast infiltration in gastric cancer. Correlation of the THBS1, THBS2, INHBA, CXCL12, TGFB, VEGFB, COL10A1, AREG, or EFNA5 expression with the cancer-associated fibroblast infiltration in stomach adenocarcinoma (STAD). TIDE algorithm was used to analyze TCGA STAD data in TIMER2.0. Figure S3. KM-Survival Curve for COL1A2 in stomach adenocarcinoma. Analysis was performed on UALCAN using TCGA data. Figure S4. The differential expression of six CAF markers in diffuse vs. intestinal subtypes of gastric cancer. The differential expression of A COL1A1, B COL1A2, C COL3A1, D COL5A1, E FN1, and F SPARC in diffuse vs. intestinal subtypes of gastric cancer and normal gastric tissues from corresponding patients (Abbreviated as “Normal tissue-Dif” for patients with diffuse gastric cancer and “Normal tissue-Int” for patients with intestinal gastric cancer) in the Asian Cancer Research Group gastric cancer dataset (GSE66229). Analysis was performed on GEO2R. Figure S5. The hazard ratio for CAF infiltration with respect to tumor stage in stomach adenocarcinoma. Bars indicate a 95% confidence interval for hazard ratios. TIDE algorithm was used to allocate TCGA stomach adenocarcinoma samples to high vs. low CAF infiltration groups in TIMER2.0. (* p < 0.05, *** p < 0.001). Figure S6. The interacting partners of ITGA4. Network representation for interacting partners of ITGA4 with respect to A protein types and B biological processes involved. To visualize the interacting partners of ITGA4, inBio Discover™ by Intomics A/S was used (https://inbio-discover.com/) (Intomics A/S has not endorsed the results of the published article). Figure S7. The differential expression of cancer-associated fibroblast poor prognostic signature genes in other cancers. Dif [file 12885_2022_9736_MOESM2_ESM.docx]

**ADDITIONAL FILE 2:**

**SUPPLEMENTARY FIGURES**


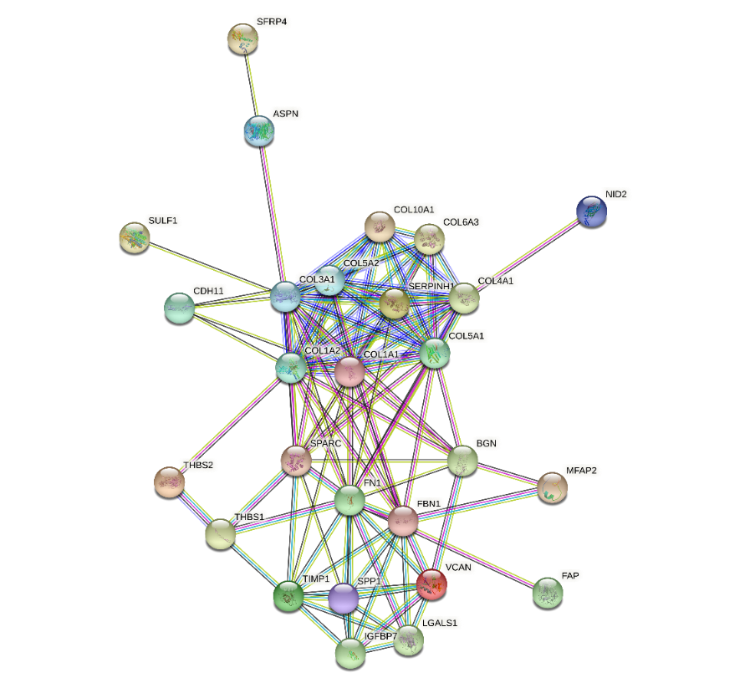


**Figure S1.** The protein-protein interaction network of the upregulated genes in gastric cancer. Disconnected nodes are hidden in the network.


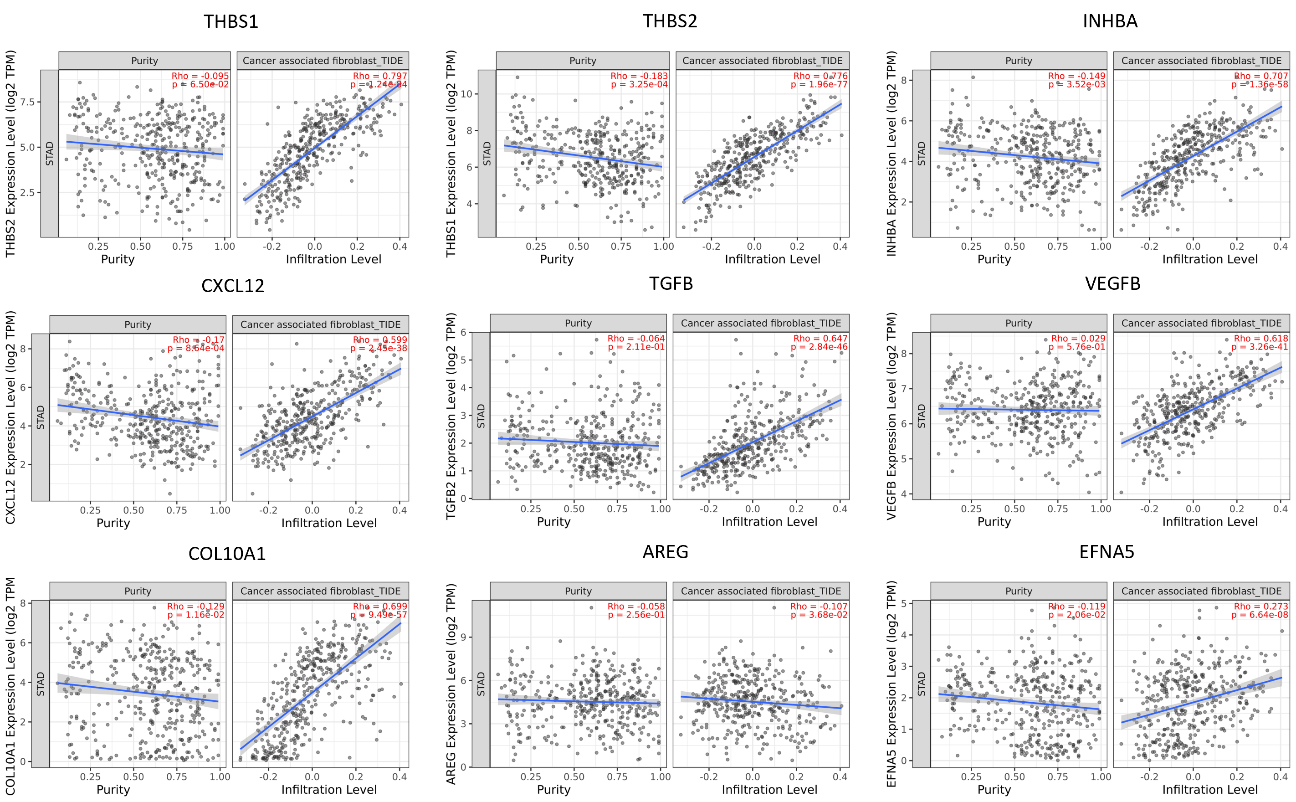


**Figure S2.** Correlation of the poor prognostic genes with cancer-associated fibroblast infiltration in gastric cancer. Correlation of the *THBS1, THBS2, INHBA, CXCL12, TGFB, VEGFB, COL10A1, AREG,* or *EFNA5* expression with the cancer-associated fibroblast infiltration in stomach adenocarcinoma (STAD). TIDE algorithm was used to analyze TCGA STAD data in TIMER2.0.


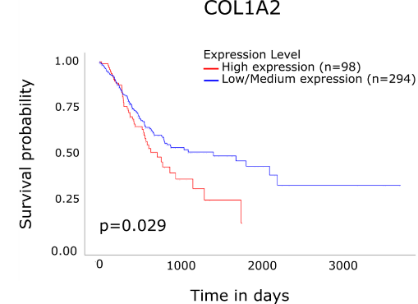


**Figure S3.** KM-Survival Curve for *COL1A2* in stomach adenocarcinoma. Analysis was performed on UALCAN using TCGA data.


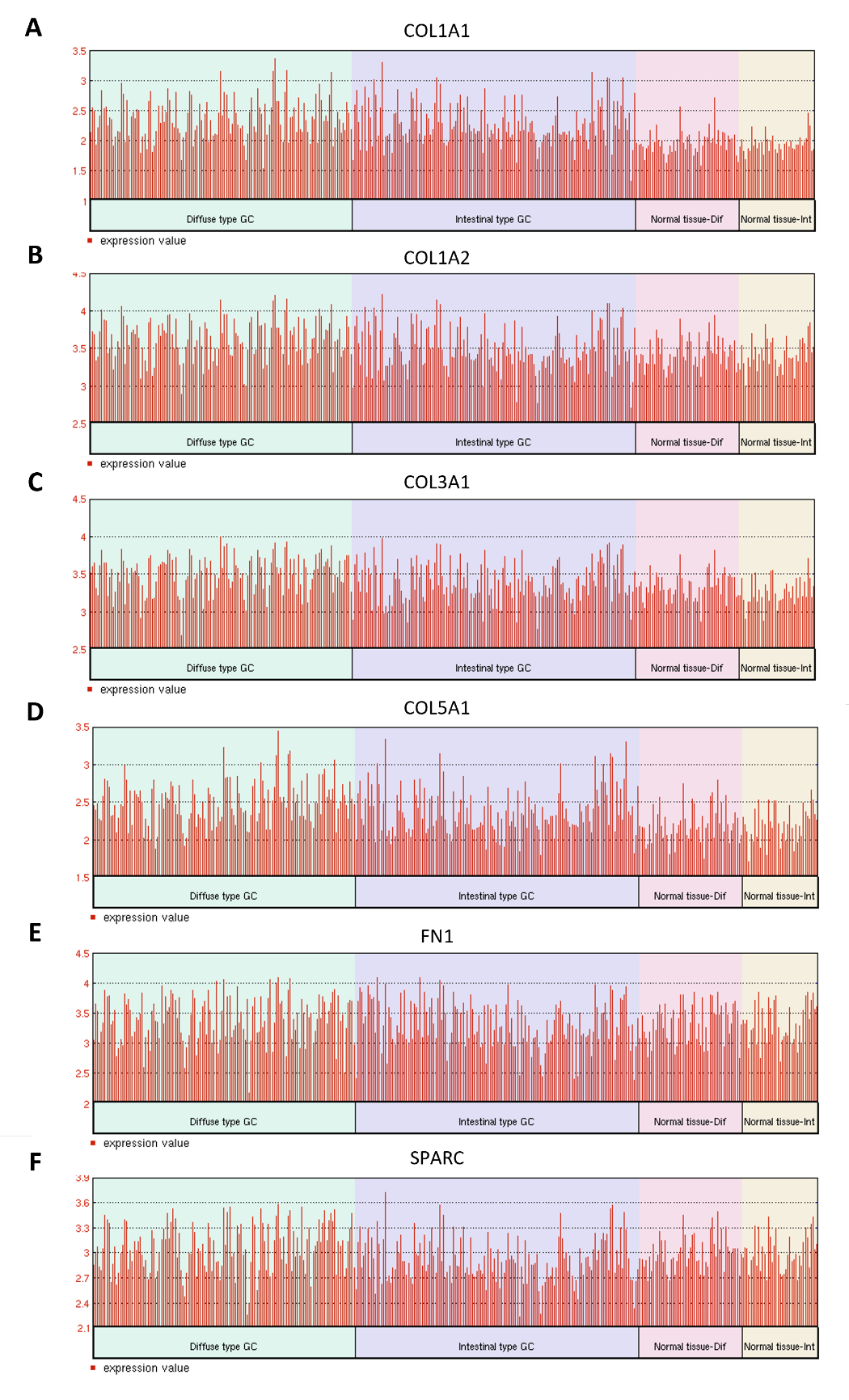


**Figure S4.** The differential expression of six CAF markers in diffuse vs. intestinal subtypes of gastric cancer. The differential expression of **A** *COL1A1*, **B** *COL1A2*, **C** *COL3A1*, **D** *COL5A1*, **E** *FN1*, and **F** *SPARC* in diffuse vs. intestinal subtypes of gastric cancer and normal gastric tissues from corresponding patients (Abbreviated as “Normal tissue-Dif” for patients with diffuse gastric cancer and “Normal tissue-Int” for patients with intestinal gastric cancer) in the Asian Cancer Research Group gastric cancer dataset (GSE66229). Analysis was performed on GEO2R.


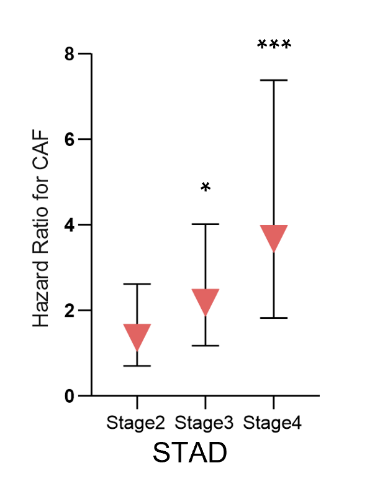


**Figure S5.** The hazard ratio for CAF infiltration with respect to tumor stage in stomach adenocarcinoma. Bars indicate a 95% confidence interval for hazard ratios. TIDE algorithm was used to allocate TCGA stomach adenocarcinoma samples to high vs. low CAF infiltration groups in TIMER2.0. (* p<0.05, *** p<0.001).


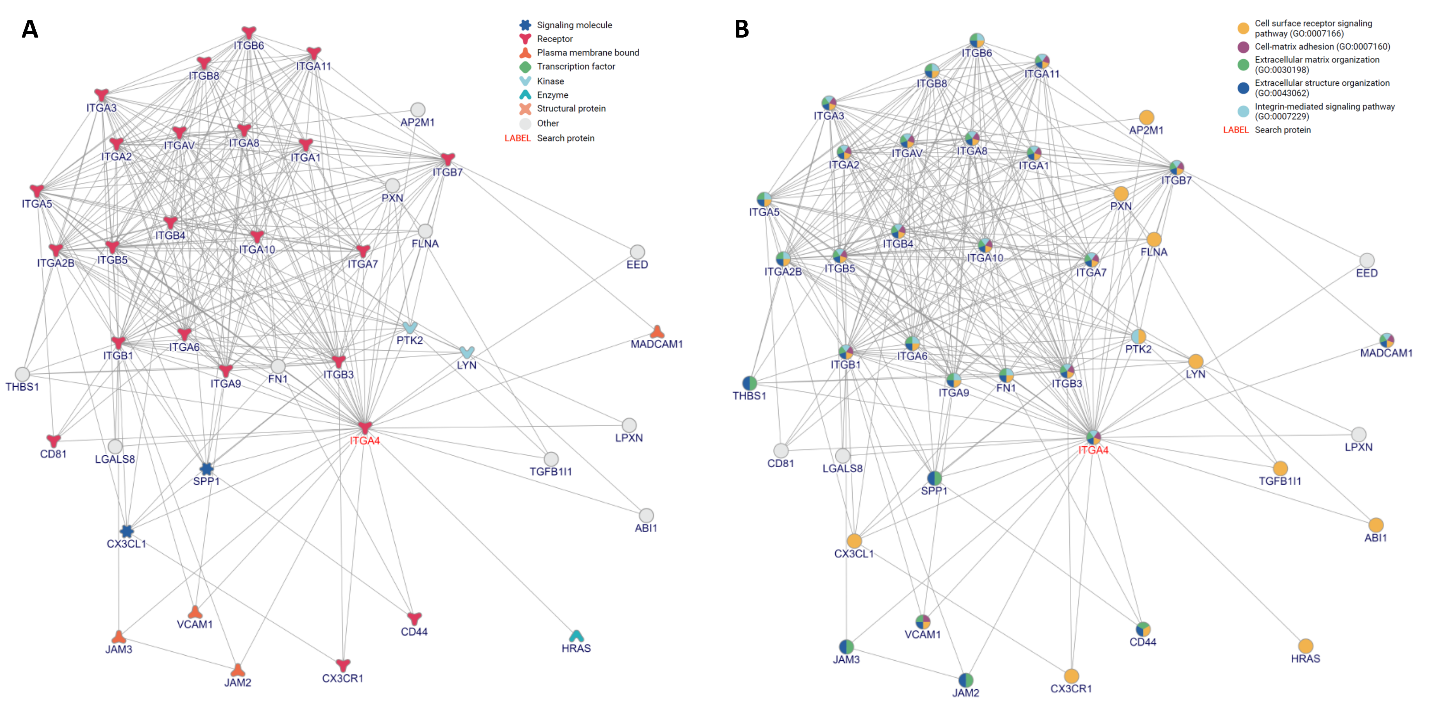


**Figure S6.** The interacting partners of ITGA4. Network representation for interacting partners of ITGA4 with respect to **A** protein types and **B** biological processes involved. To visualize the interacting partners of ITGA4, inBio Discover^TM^ by Intomics A/S was used (<https://inbio-discover.com/>) (Intomics A/S has not endorsed the results of the published article).


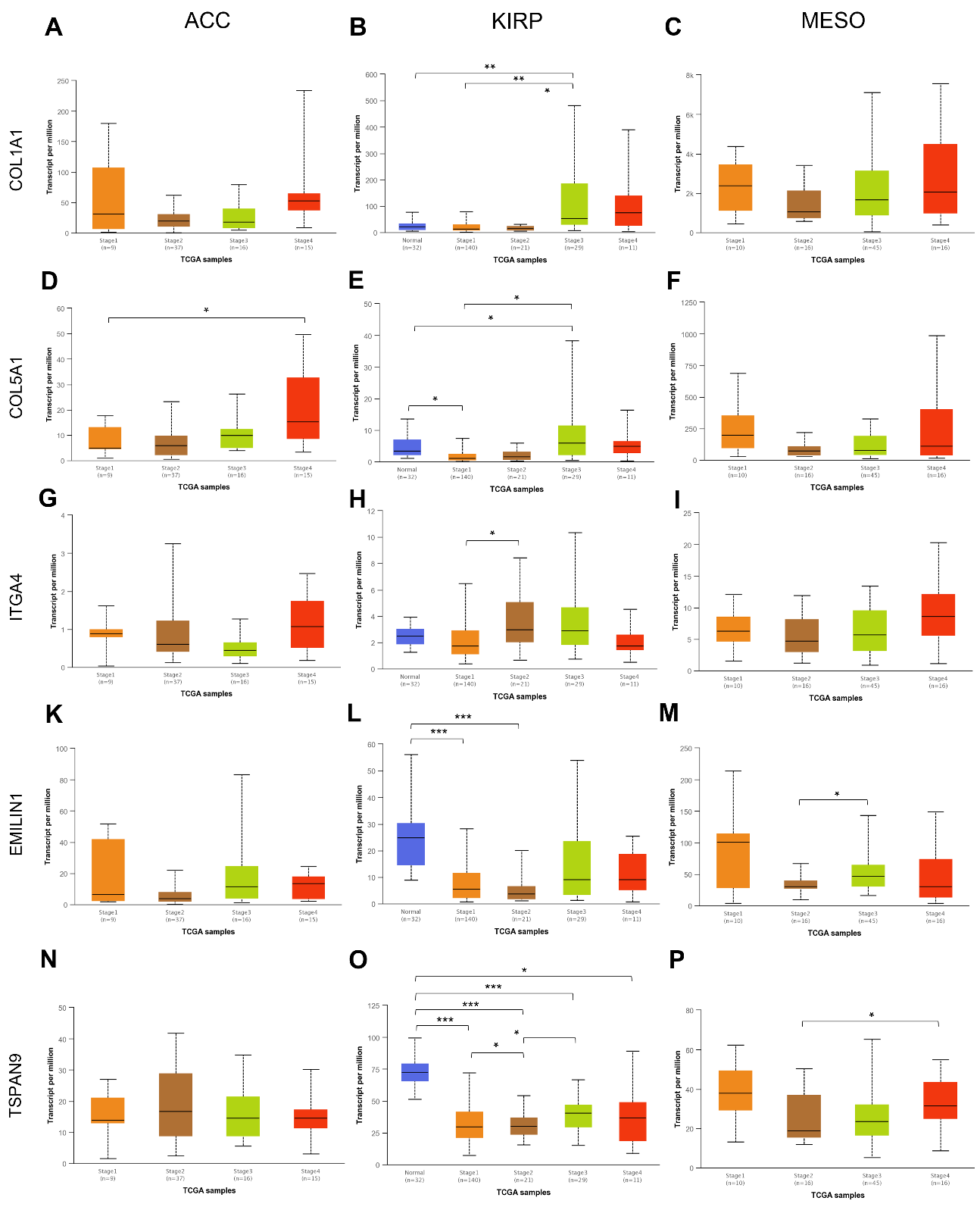


**Figure S7.** The differential expression of cancer-associated fibroblast poor prognostic signature genes in other cancers. Differential expression of **A-C** *COL1A1*, **D-F** *COL5A1*, **G-I** *ITGA4,* **K-M** *EMILIN1,* and **N-P** *TSPAN9* with respect to tumor stage in adrenocortical carcinoma (ACC), kidney renal papillary cell carcinoma (KIRP), and mesothelioma (MESO). TCGA data was analyzed on UALCAN (unpaired t-test, * p<0.05, ** p<0.01, *** p<0.001).


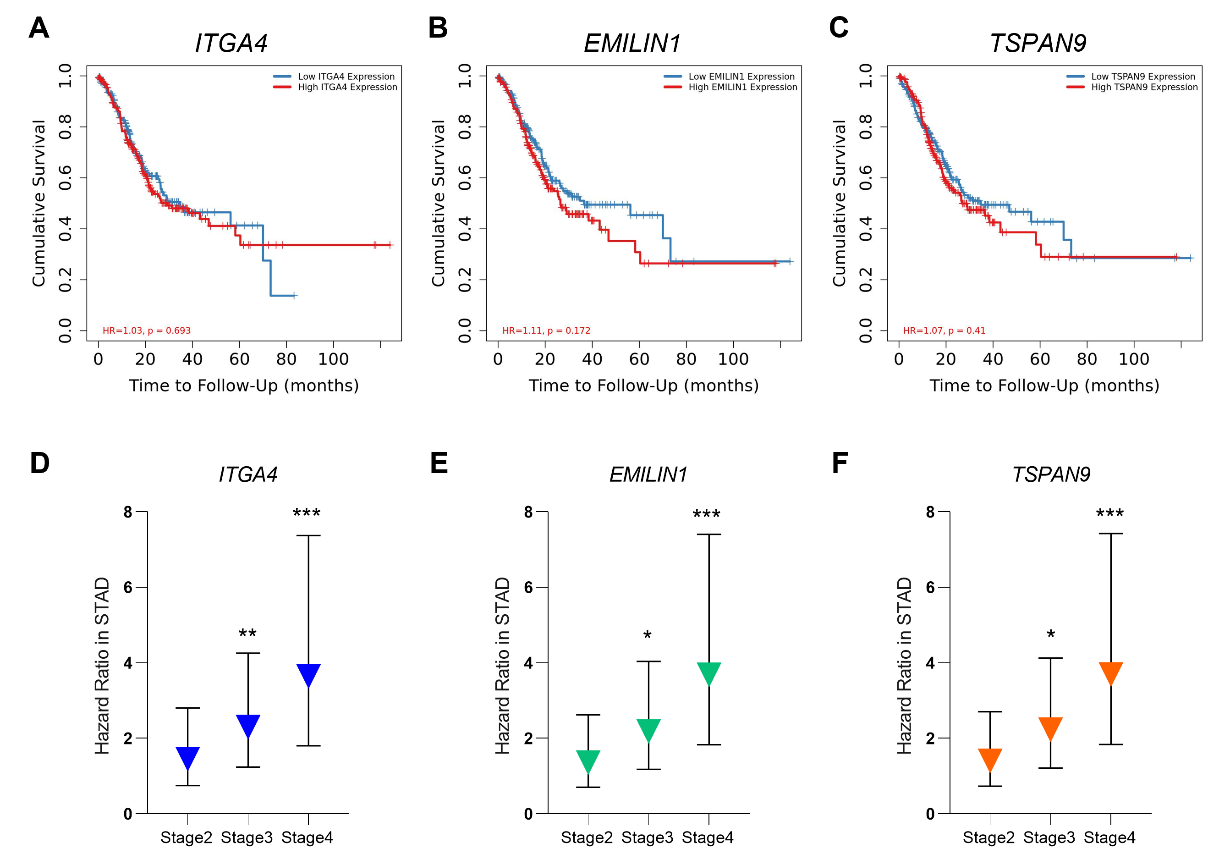


**Figure S8.** The prognostic impact of *ITGA4, EMILIN1,* and *TSPAN9* in gastric cancer. Kaplan-Meier survival curves for **A** *ITGA4,* **B** *EMILIN1,* and **C** *TSPAN9* in gastric cancer. The increase in the hazard ratio in the Cox proportional regression model for **D** *ITGA4,* **E** *EMILIN1,* and **F** *TSPAN9* by stage in gastric cancer. Bars indicate the 95% confidence interval for hazard ratios. TCGA stomach adenocarcinoma samples were analyzed in TIMER2.0. (* p<0.05, ** p<0.01, *** p<0.001).
